# Supplementary material for: Early disruption of the innate-adaptive immune axis in vivo after infection with virulent Georgia 2007/1 ASFV
Source: Discov Immunol. 2025 Oct 23;4(1):kyaf014. doi: 10.1093/discim/kyaf014 (PMC12775366; doi:10.1093/discim/kyaf014)
Supplement: kyaf014_Supplementary_Data [file kyaf014_supplementary_data.pdf]

Early disruption of the innate-adaptive immune axis *in vivo* after infection with virulent Georgia2007/1 ASFV

Priscilla YL Tng*, Laila Al-Adwani, Lynnette Goatley, Raquel Portugal, Anusyah Rathakrishnan, Christopher L Netherton*

* Corresponding authors

Supplementary Data

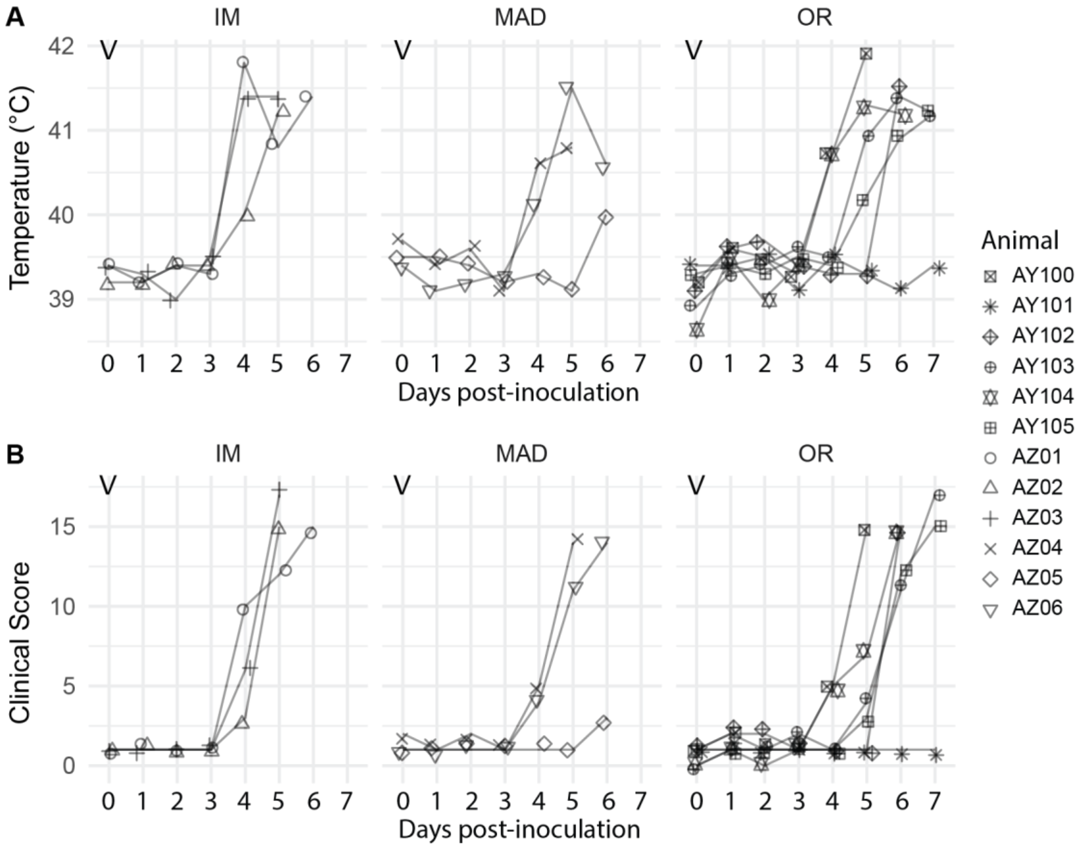

Supplementary Figure 1 (A) Temperatures and (B) clinical scores of outbred domestic pigs inoculated with Georgia2007/1 with different methods: intramuscular injection (IM), intranasally with a MAD device (MAD) and oronasally (OR). Each datapoint denotes a single animal. Time of virus inoculation is indicated by the arrowhead.

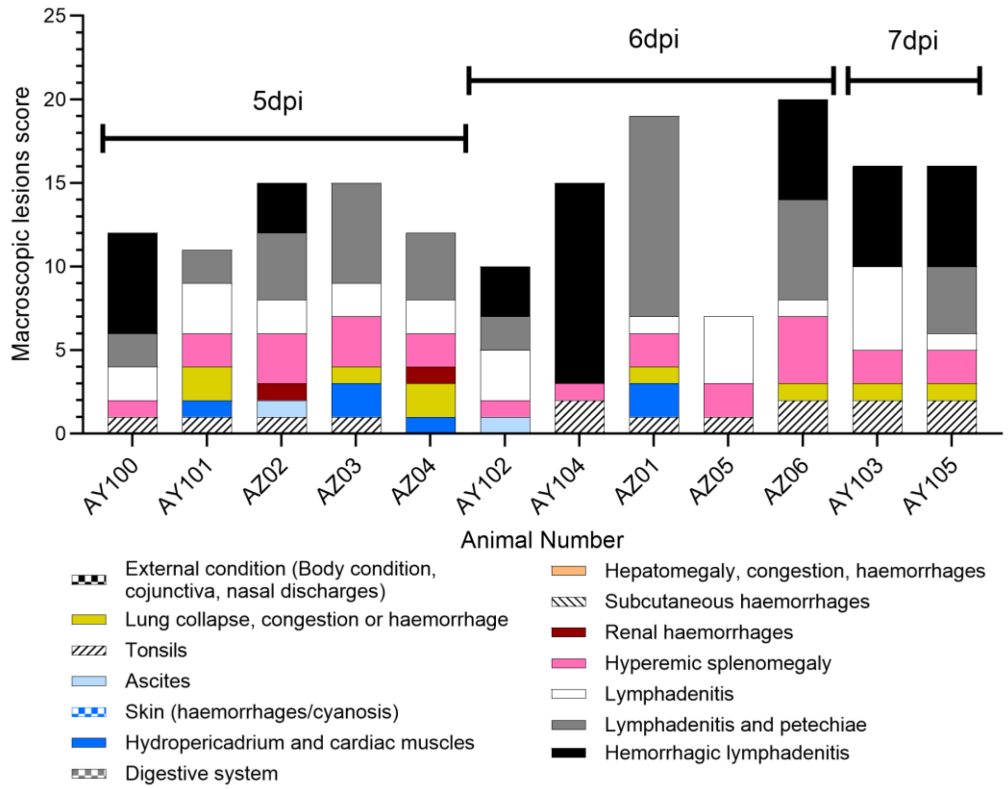

Supplementary Figure 2 Macroscopic scoring of outbred domestic pigs inoculated with Georgia2007/1 with different methods: intramuscular injection (IM) (AZ01 – AZ03), intranasally with a MAD device (AZ04 – AZ06) and oronasally (AY100 – AY105). Each bar represents the total lesion score for each animal and the scores of each tissue are denoted by different colours within the bar. The animal numbers are listed below each bar and the animals are grouped by the day they were euthanised after inoculation. Necropsy was performed on the day of euthanasia and macroscopic lesions were evaluated as described by Galindo-Cardiel et al., 2013. dpi: days after inoculation with Georgia 2007/1.

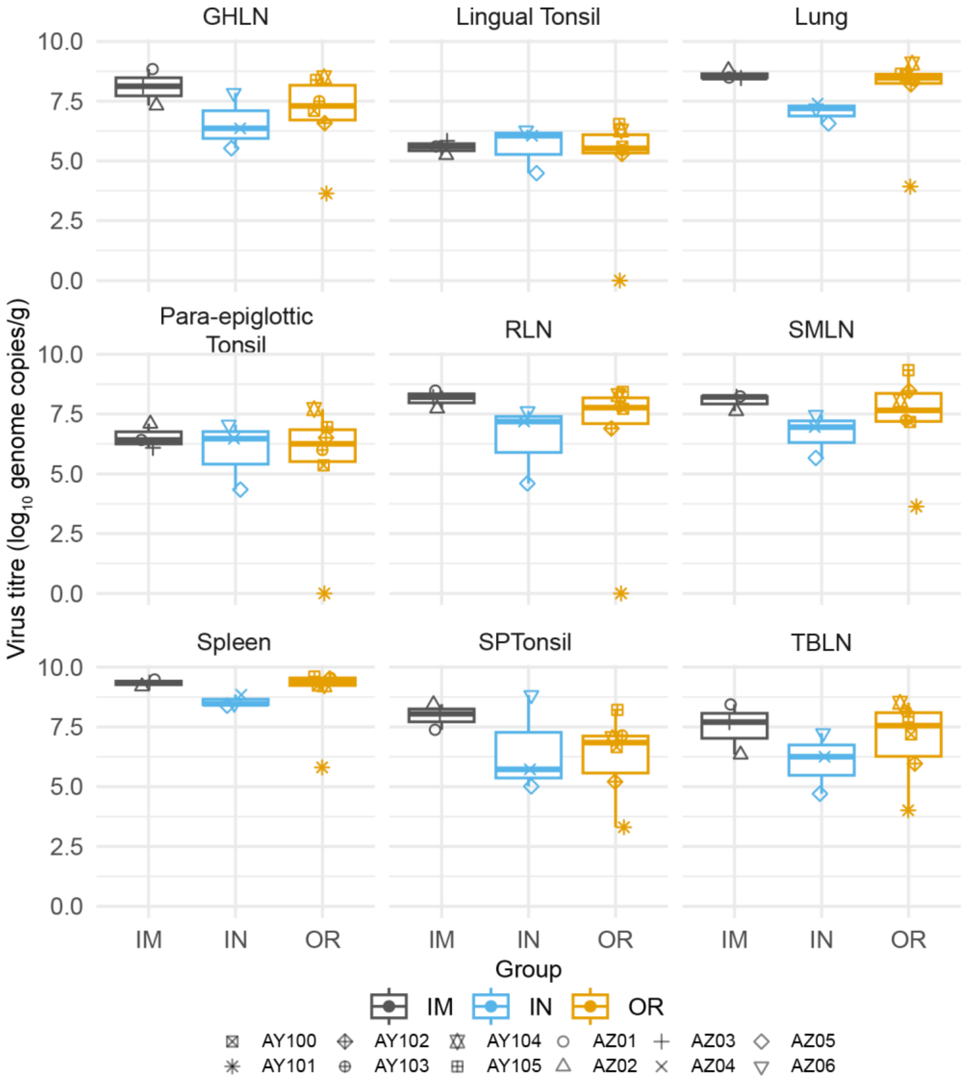

Supplementary Figure 3 Viral load in tissues of outbred animals euthanised when they reached humane endpoints after inoculation with Georgia2007/1 with different methods: intramuscular injection (IM) (AZ01 – AZ03), intranasally (IN) with a MAD device (AZ04 – AZ06) and oronasally (OR) (AY100 – AY105). Each datapoint denotes a single animal. (TBLN, GHLN) ANOVA, (other tissues) Kruskal-Wallis. GHLN: gastro-hepatic lymph node, RPLN: retropharyngeal lymph node, RLN: renal lymph node, SMLN: submandibular lymph node, SPTonsil: soft palate tonsil, TBLN: tracheal bronchial lymph node.

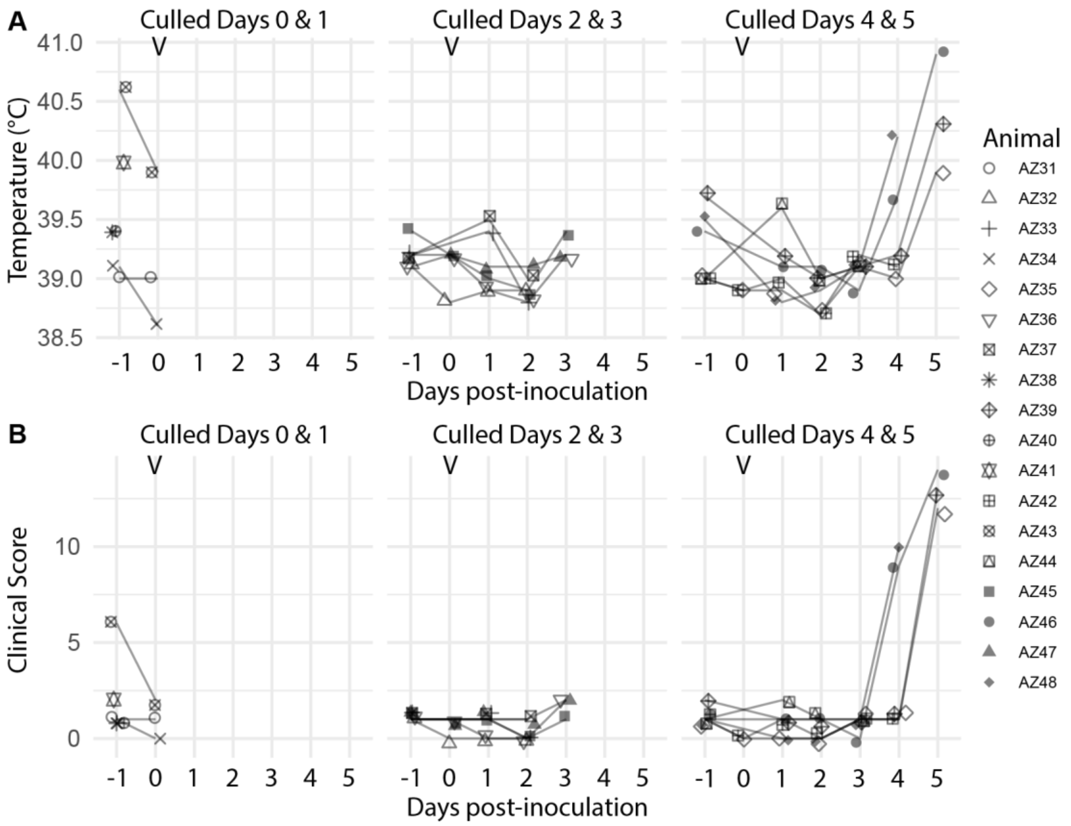

Supplementary Figure 4 (A) Temperatures and (B) clinical scores of inbred Babraham pigs culled in the sequential cull experiment grouped as animals culled on days 0 and 1, days 2 and 3, and days 4 and 5 after oronasal inoculation with Georgia2007/1 (indicated by arrowhead).

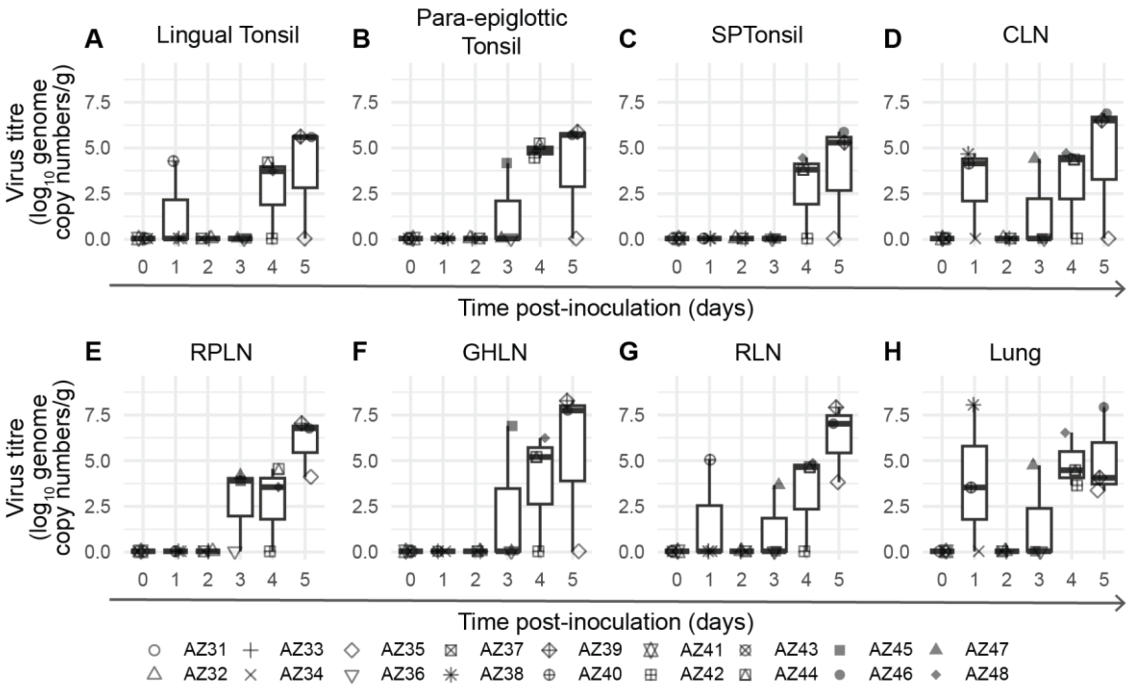

Supplementary Figure 5 Viral load in tissues of animals oronasally inoculated with Georgia2007/1 and sequentially culled between 0 – 5 dpi. Each datapoint denotes a single animal. Kruskal-Wallis except for RLN which used one-way ANOVA. CLN: cervical lymph node, GHLN: gastro-hepatic lymph node, RLN: renal lymph node, RPLN: retropharyngeal lymph node, SPTonsil: soft palate tonsil.

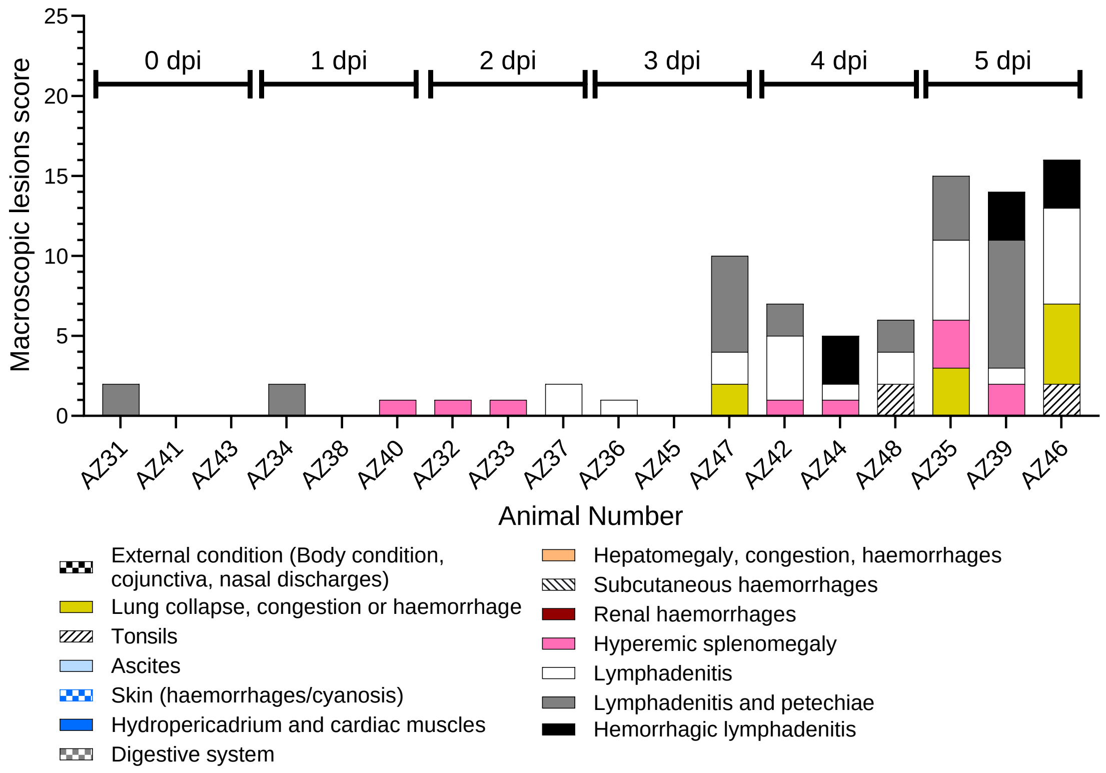

Supplementary Figure 6 Macroscopic scoring of animals in the sequential cull experiment. Each bar represents the total lesion score for each animal and the scores of each tissue are denoted by different colours within the bar. The animal numbers are listed below each bar, and the Animals are grouped by the day they were euthanised after inoculation with Georgia2007/1. Necropsy was

performed on the day of euthanasia and macroscopic lesions were evaluated as described by Galindo-Cardiel et al., 2013. dpi: days post-inoculation with Georgia 2007/1.

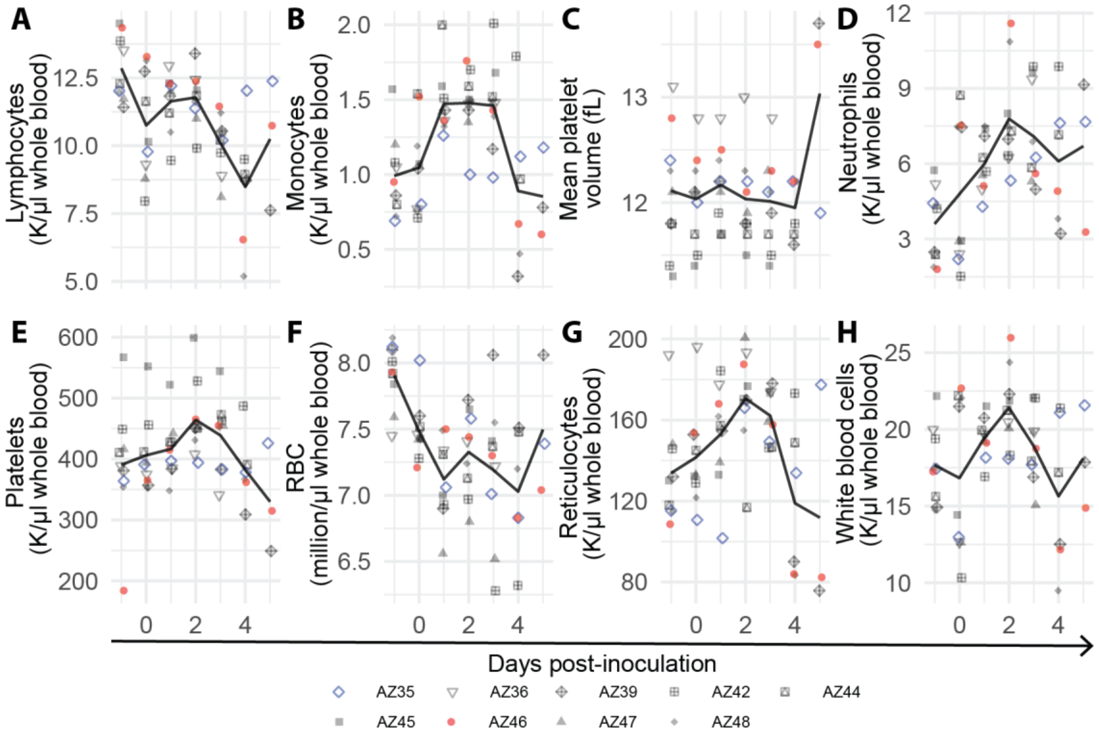

Supplementary Figure 7 Haematological measurements with the IDEXX, red points: animal AZ46 which reached its humane endpoints on 5 dpi, blue points: animal AZ35 which had no detectable viraemia up to 5 dpi. Each datapoint denotes a single animal and the black line denotes the mean.

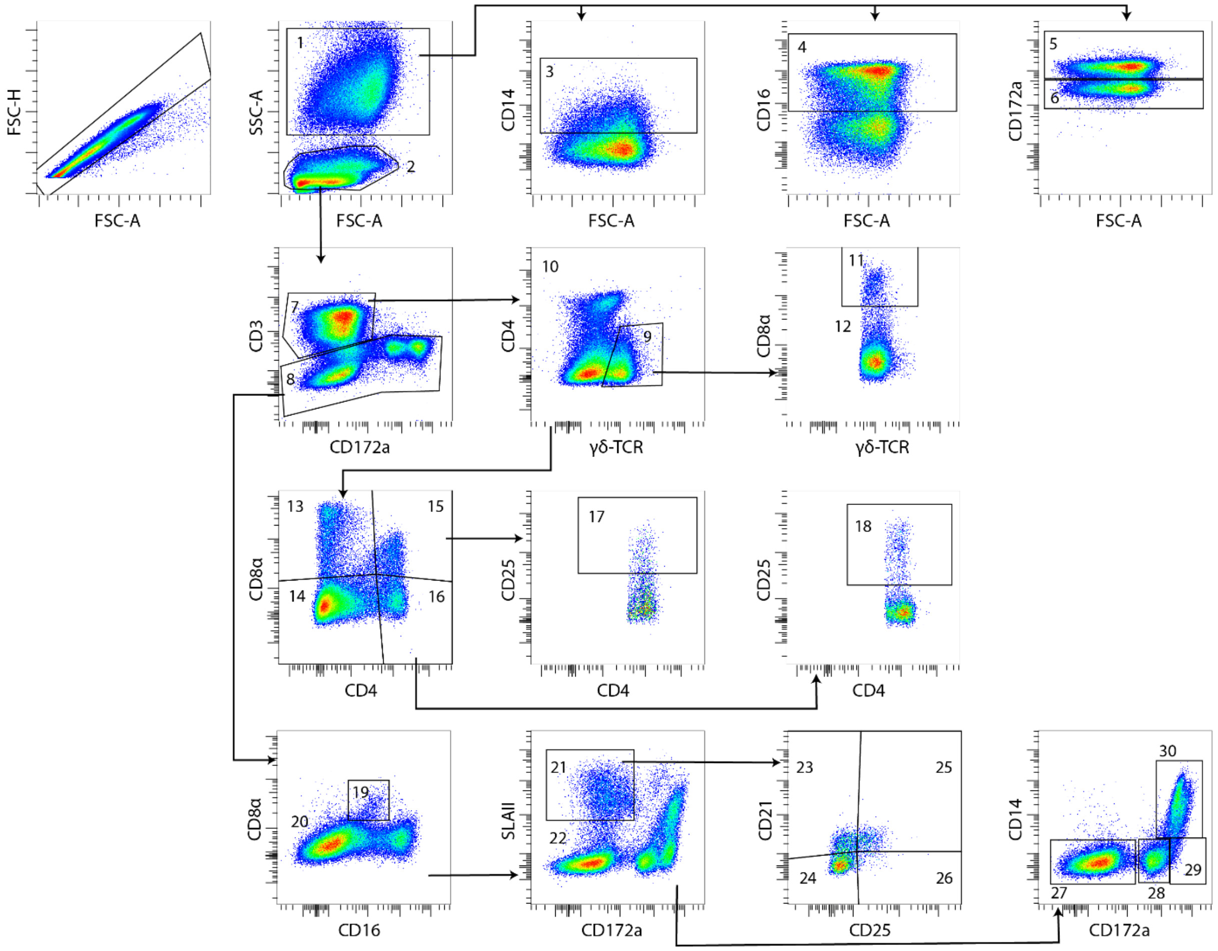

Supplementary Figure 8 Gating strategy for volumetric whole blood staining. Representative gating of whole blood sample from AZ33 at 2 dpi. (1, 3 - 6) Granulocytes, (2) monocytes and lymphocytes, (7) CD3^{+} T cells, (9) γδ-TCR^{+} T cells, (10) γδ-TCR^{-} T cells, (11) CD8α^{+} γδ-TCR^{+} T cells, (13) CTLs and CD4^{-}CD8α^{+}NKT, (14) unconventional CD4-CD8α^{-} T cells, (15) CD4^{+}CD8α^{+} activated T-helper and Treg cells, (16) naïve CD4^{+}CD8α^{-} T-helper and Treg cells, (17) CD4^{+}CD8α^{+}CD25^{+} Treg containing population, (18) CD4^{+}CD8α^{-}CD25^{+} Treg containing population, (19) NK cells, (21) SLAII^{+}CD172a$^{-/lo}$, (23) CD21^{+}CD25^{-} B cells, (24) SLAII^{+}CD172a$^{-/lo}$CD21^{-}CD25^{-}, (25) CD21^{+}CD25^{+} B cells, (26) SLAII^{+}CD172a$^{-/lo}$CD21^{-}CD25^{+}, (27) CD172a^{-}, (28) CD172a^{+}CD14^{-}, (29) CD172a^{++}CD14^{-}, and (30) CD172a^{++}CD14^{+} monocytes.

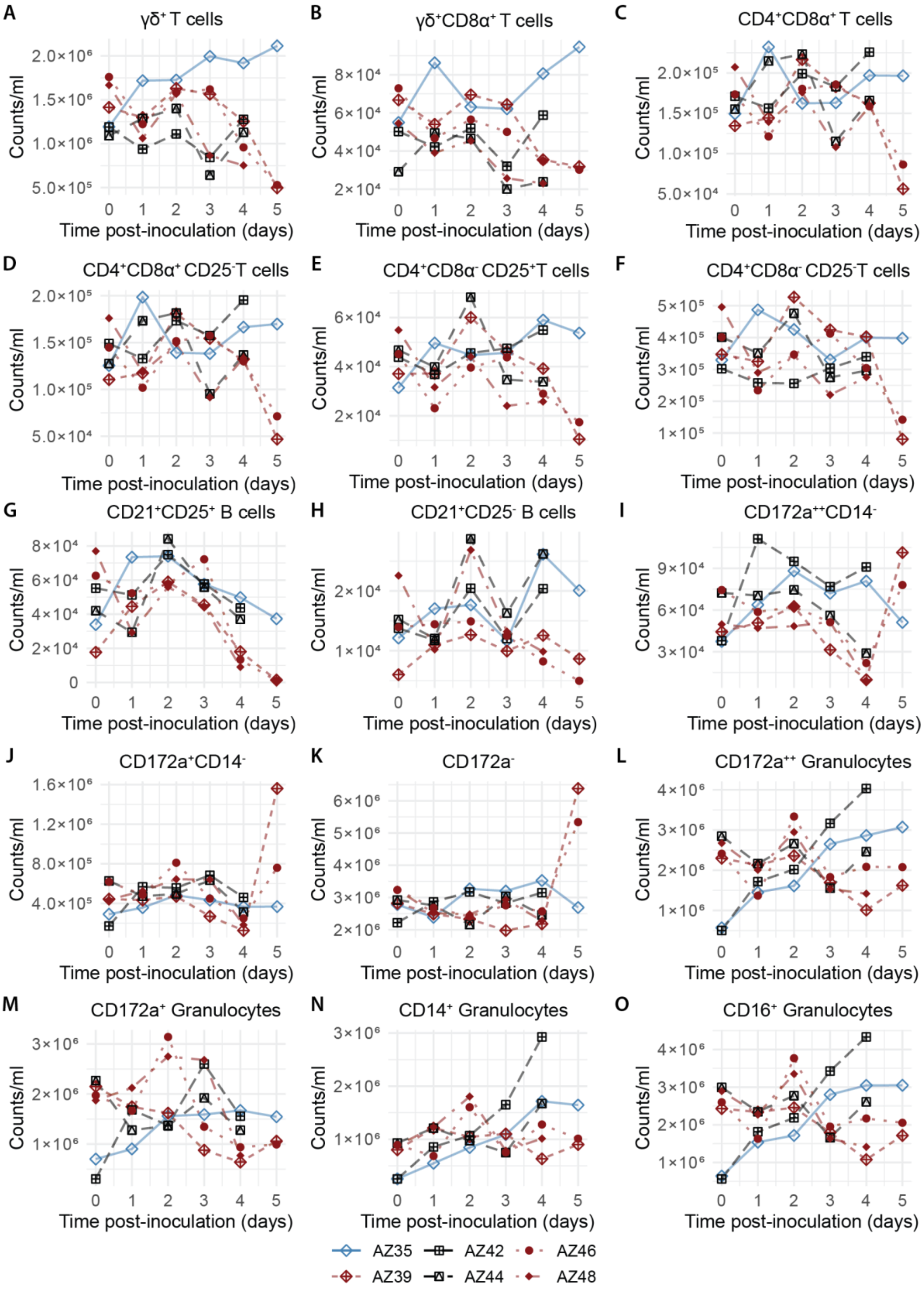

Supplementary Figure 9 Dynamics of immune cells within whole blood post-inoculation with Georgia 2007/1, as determined with volumetric flow cytometry. Each datapoint denotes a single animal. The animal (AZ35) that was not viraemic on 5 dpi is denoted in blue and the animals with the most severe disease (AZ39, AZ46 and AZ48) are denoted in red. Repeated-measures linear mixed effect model, except for G, J, L, M, N, and O where linear model was used due to the absence of random effects.

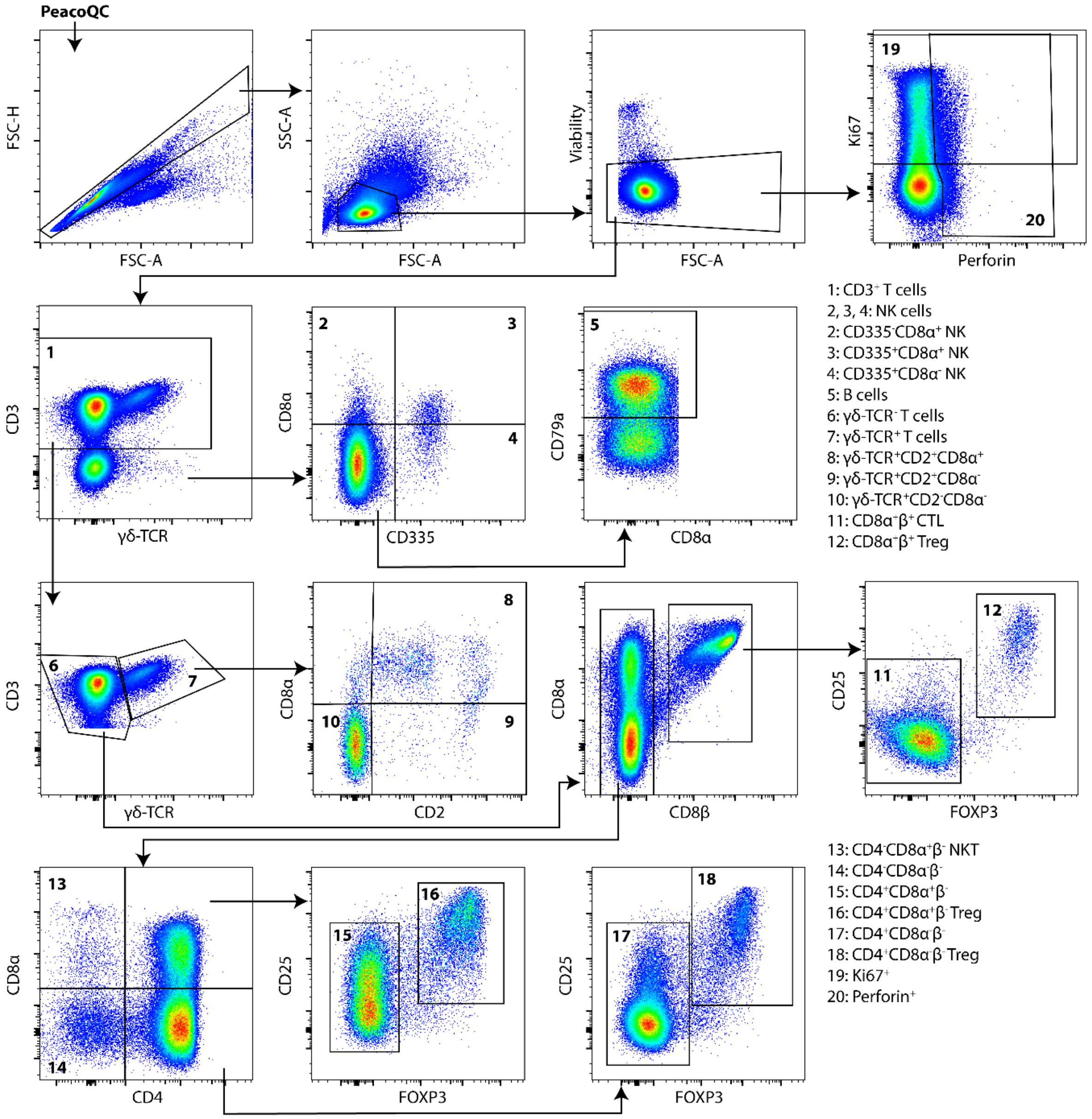

Supplementary Figure 10 Gating strategy for (1) CD3^{+} T cell tSNE and conventional gating analysis. Representative gating of SMLN sample from AZ36 on day 3 post-inoculation. (2-4) NK cells, (5) B cells, (6) γδ-TCR^{-} T cells, (7) γδ-TCR^{+} T cells, (8) effector γδ-TCR^{+} T cells, (9) activated γδ-TCR^{+} T cells, (10) naïve γδ-TCR^{+} T cells, (11) CTLs, (12) CD4^{-}CD8α^{+}β^{+} Tregs, (13) CD4^{-}CD8α^{+}β^{-} NKT, (14) unconventional CD4^{-}CD8α^{-}β^{-} T cells, (15) CD4^{+}CD8α^{+}β^{-} DP T-helper cells, (16) CD4^{+}CD8α^{+}β^{-} Tregs, (17) naïve CD4^{+}CD8α^{-}β^{-} T-helper cells, and (18) CD4^{+}CD8α^{-}β^{-} Treg containing populations. Subsequent Boolean gating with (19) provide frequencies of proliferating cells and Boolean gating with (20) provide frequencies of cells expressing perforin.

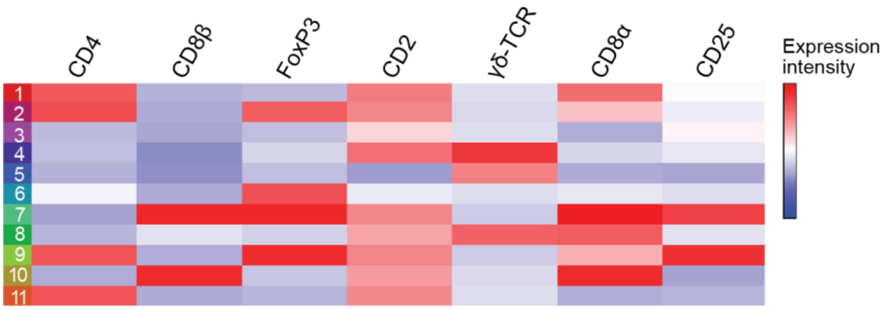

Supplementary Figure 11 Heatmap of clusters identified by FlowSOM on CD3$^+$ cells subjected to tSNE analysis and the associated marker expression profiles. Numbers on the left column indicate the clusters. Cluster 1: CD4$^+$CD8α$^+$β$^-$ DP T-helper cells, cluster 2: CD4$^+$CD8α$^+$FoxP3$^+$ T cells, cluster 3: unconventional CD4$^-$CD8α$^-$ T cells, activated γδ-TCR$^+$ T cells, cluster 4: activated γδ-TCR$^+$ T cells, cluster 5: naïve γδ-TCR$^+$ T cells, cluster 6: unconventional CD4$^-$CD8α$^-$FoxP3$^+$ T cells, cluster 7: CD4$^-$CD8α$^+$β$^+$CD25$^+$FoxP3$^+$ Tregs, cluster 8: effector γδ-TCR$^+$ T cells, cluster 9: CD4$^+$CD8α$^+$β$^-$CD25$^+$FoxP3$^+$ Tregs, cluster 10: CTLs, cluster 11: naïve CD4$^+$CD8α$^-$β$^-$ T-helper cells.

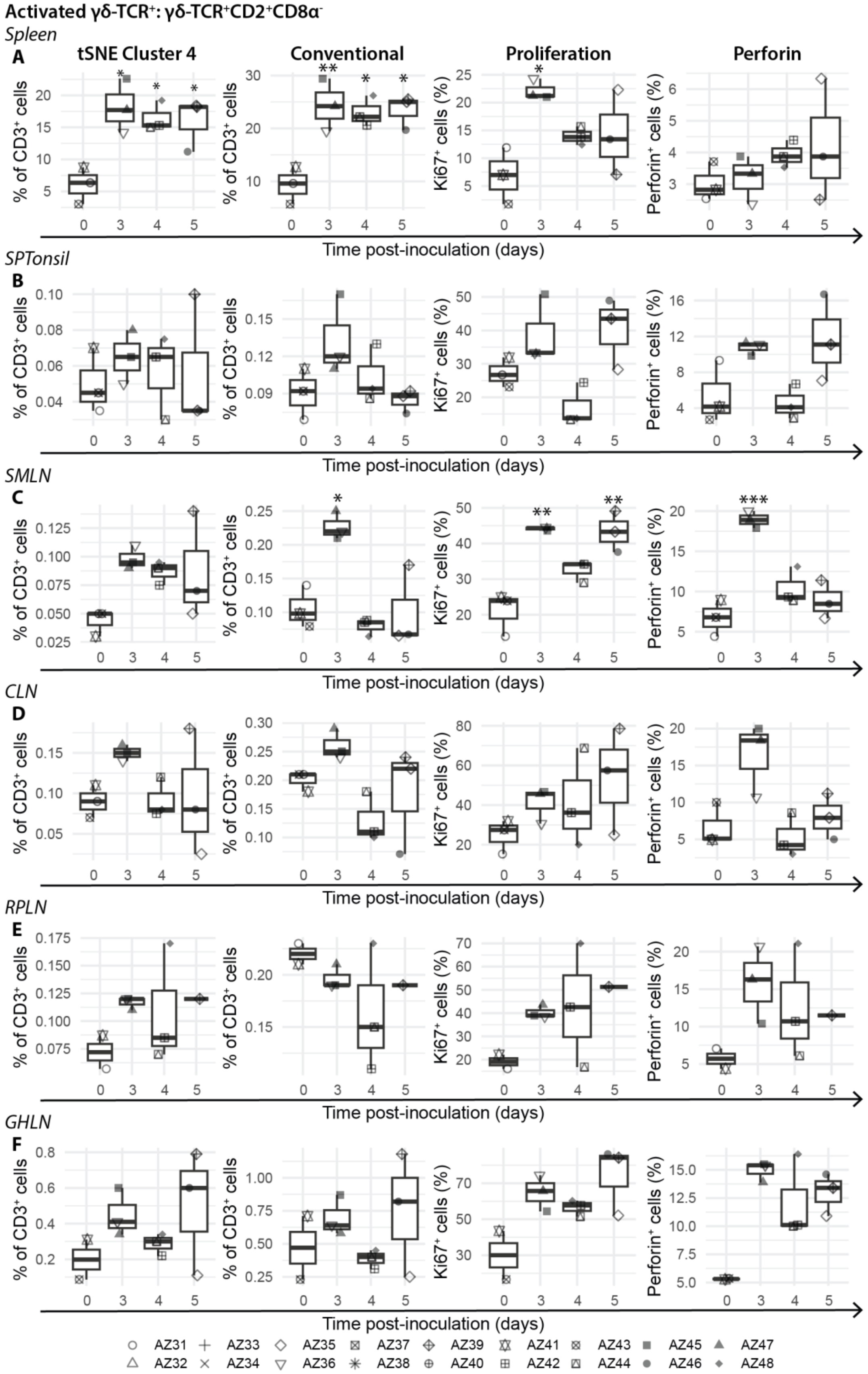

Supplementary Figure 12 Dynamics, proliferation and perforin expression of CD2+CD8α-γδ-TCR+ cells in various tissues post-inoculation with Georgia 2007/1. Each datapoint denotes a single animal. All tissues have n=3 samples at each timepoint except for RPLN 0 dpi (n=2) and 5 dpi (n=1), and GHLN 0 dpi (n=2). CLN: cervical lymph node, GHLN: gastro-hepatic lymph node, RPLN:

retropharyngeal lymph node, SMLN: submandibular lymph node, SPTonsil: soft palate tonsil. * p<0.05, ** p<0.01, *** p<0.001, one way ANOVA.

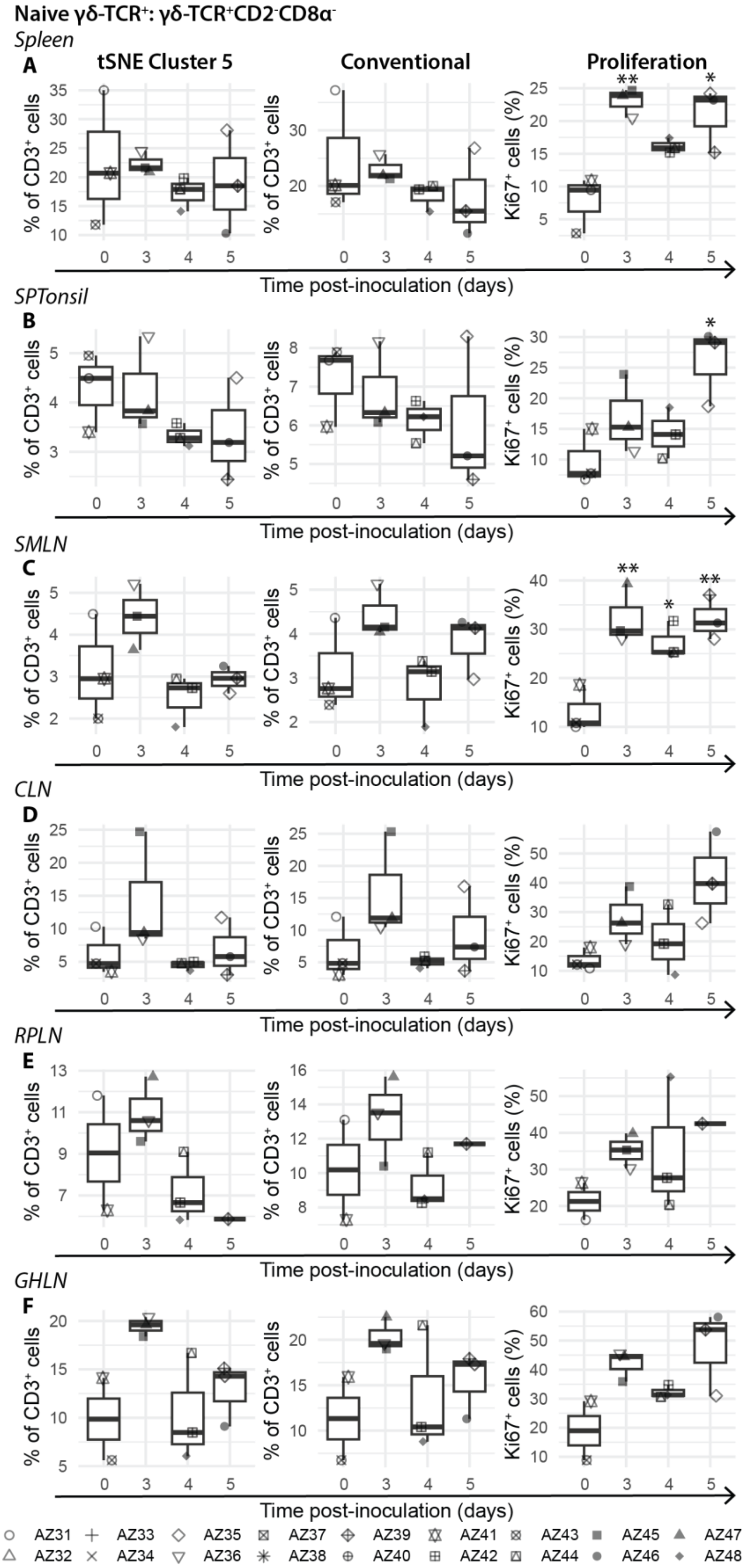

Supplementary Figure 13 Dynamics and proliferation of $CD2^-CD8\alpha^-\gamma\delta$-$TCR^+$ cells in various tissues post-inoculation with Georgia 2007/1. Each datapoint denotes a single animal. All tissues have n=3

samples at each timepoint except for RPLN 0 dpi (n=2) and 5 dpi (n=1), and GHLN 0 dpi (n=2). CLN: cervical lymph node, GHLN: gastro-hepatic lymph node, RPLN: retropharyngeal lymph node, SMLN: submandibular lymph node, SPTonsil: soft palate tonsil. * $p<0.05$, ** $p<0.01$, one way ANOVA, except for (D, tSNE Cluster 5) which used Kruskal-Wallis.

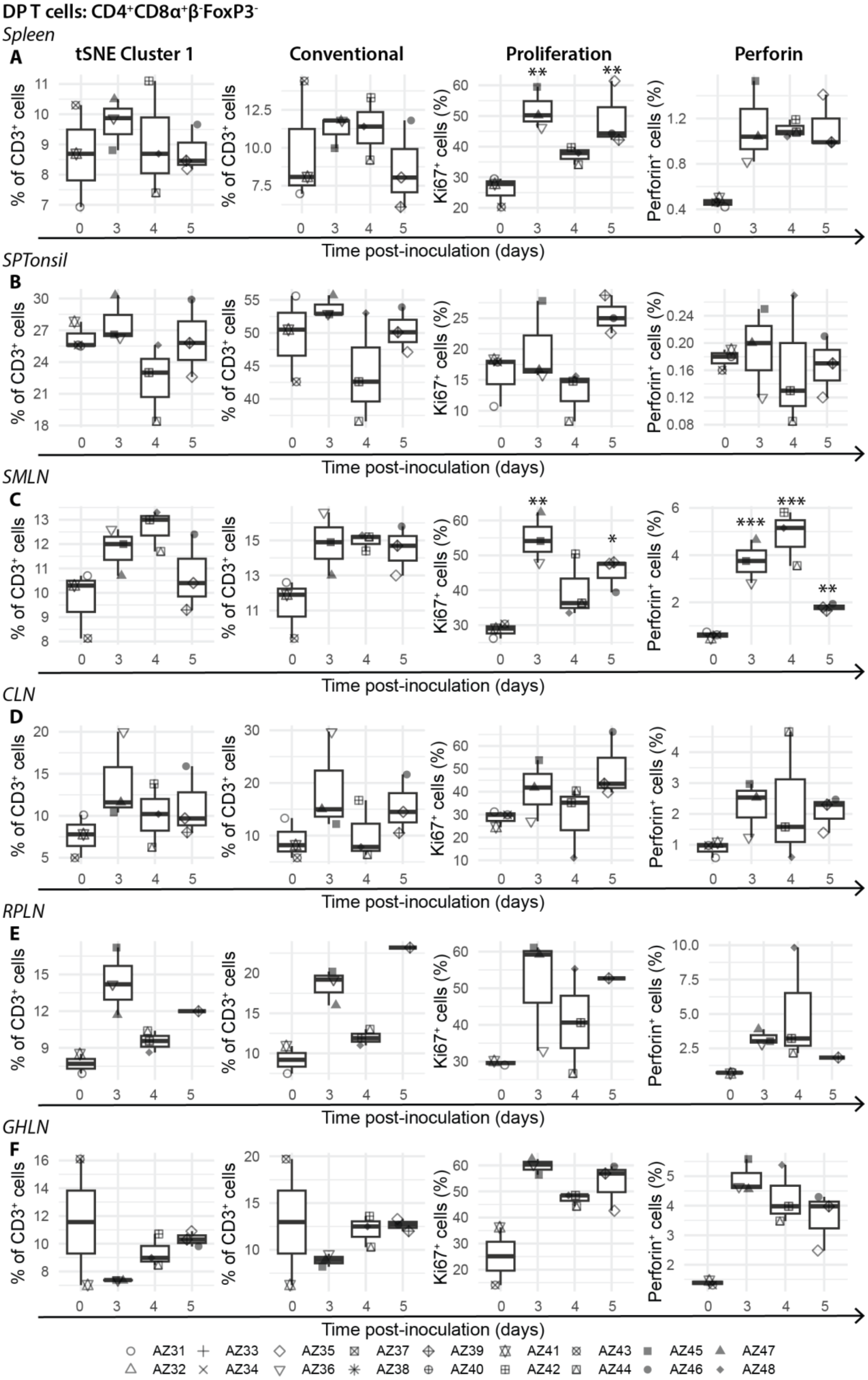

Supplementary Figure 14 Dynamics, proliferation and perforin expression of DP T cells in various tissues post-inoculation with Georgia 2007/1. Each datapoint denotes a single animal. All tissues have n=3 samples at each timepoint except for RPLN 0 dpi (n=2) and 5 dpi (n=1), and GHLN 0 dpi (n=2). CLN: cervical lymph node, GHLN: gastro-hepatic lymph node, RPLN: retropharyngeal lymph node, SMLN: submandibular lymph node, SPTonsil: soft palate tonsil. * $p<0.05$, ** $p<0.01$, one way ANOVA, except for (A, Perforin) and (C, Conventional) which used Kruskal-Wallis.

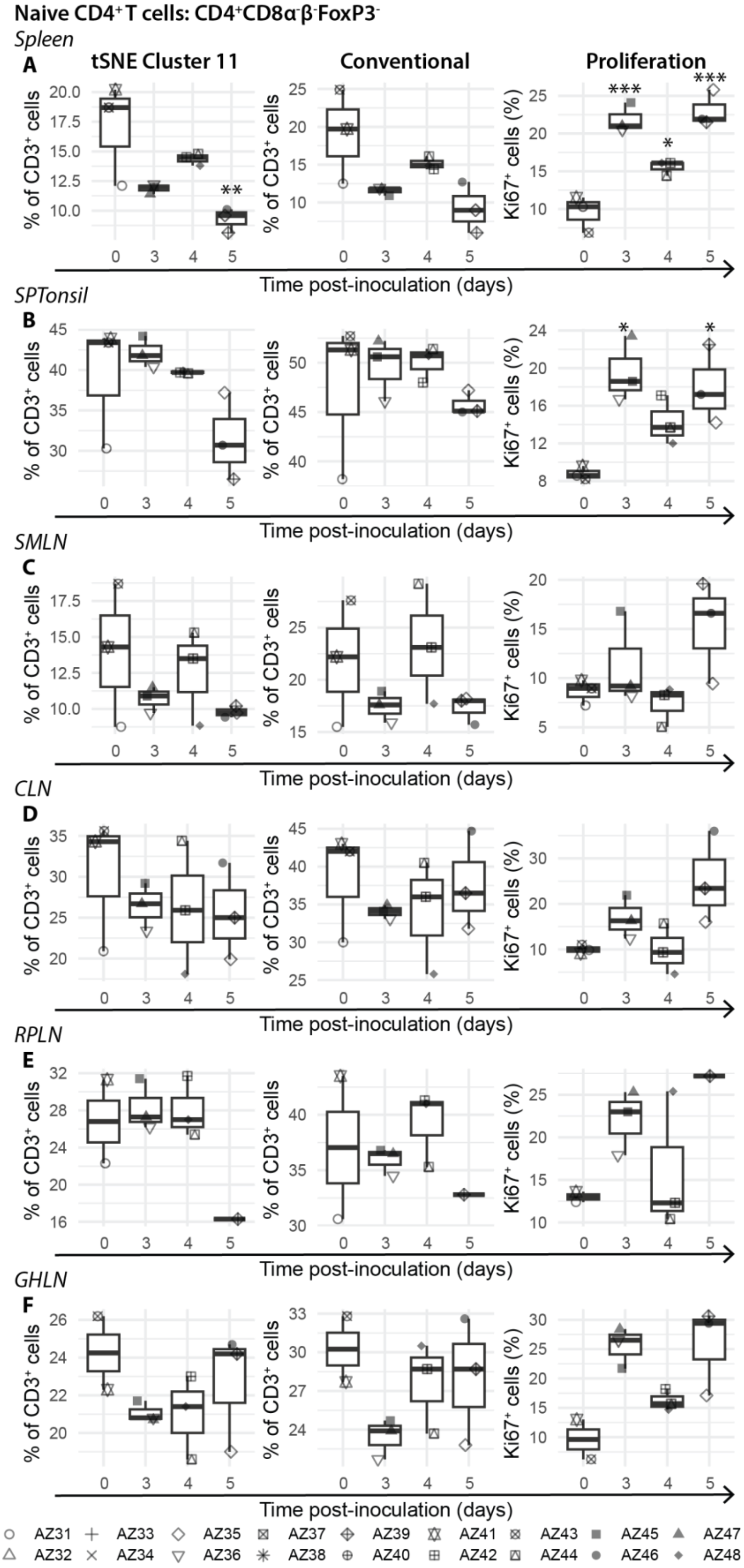

Supplementary Figure 15 Dynamics and proliferation of naïve CD4+ T cells in various tissues post-inoculation with Georgia 2007/1. Each datapoint denotes a single animal. All tissues have n=3 samples at each timepoint except for RPLN 0 dpi (n=2) and 5 dpi (n=1), and GHLN 0 dpi (n=2). CLN: cervical lymph node, GHLN: gastro-hepatic lymph node, RPLN: retropharyngeal lymph node, SMLN: submandibular lymph node, SPTonsil: soft palate tonsil. * $p<0.05$, *** $p<0.001$, one way

ANOVA, except for (B, tSNE Cluster 11, Proliferation) and (C, tSNE Cluster 11, Conventional) which used Kruskal-Wallis.

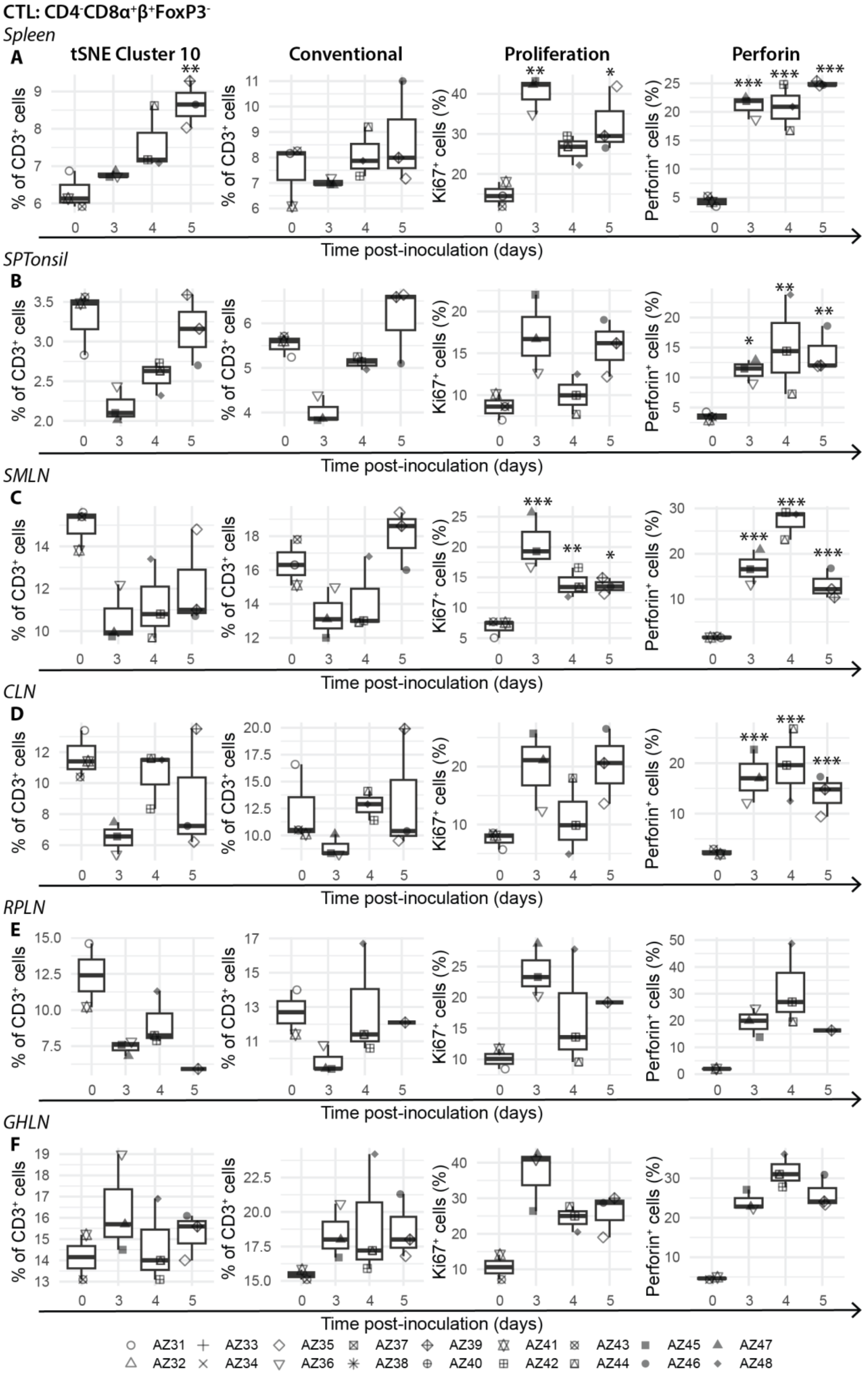

Supplementary Figure 16 Dynamics, proliferation and perforin expression of CTL cells in various tissues post-inoculation with Georgia 2007/1. Each datapoint denotes a single animal. All tissues

have n=3 samples at each timepoint except for RPLN 0 dpi (n=2) and 5 dpi (n=1), and GHLN 0 dpi (n=2). CLN: cervical lymph node, GHLN: gastro-hepatic lymph node, RPLN: retropharyngeal lymph node, SMLN: submandibular lymph node, SPTonsil: soft palate tonsil. * p<0.05, ** p<0.01, *** p<0.001, one way ANOVA, except for (A, Conventional) which used Kruskal-Wallis.

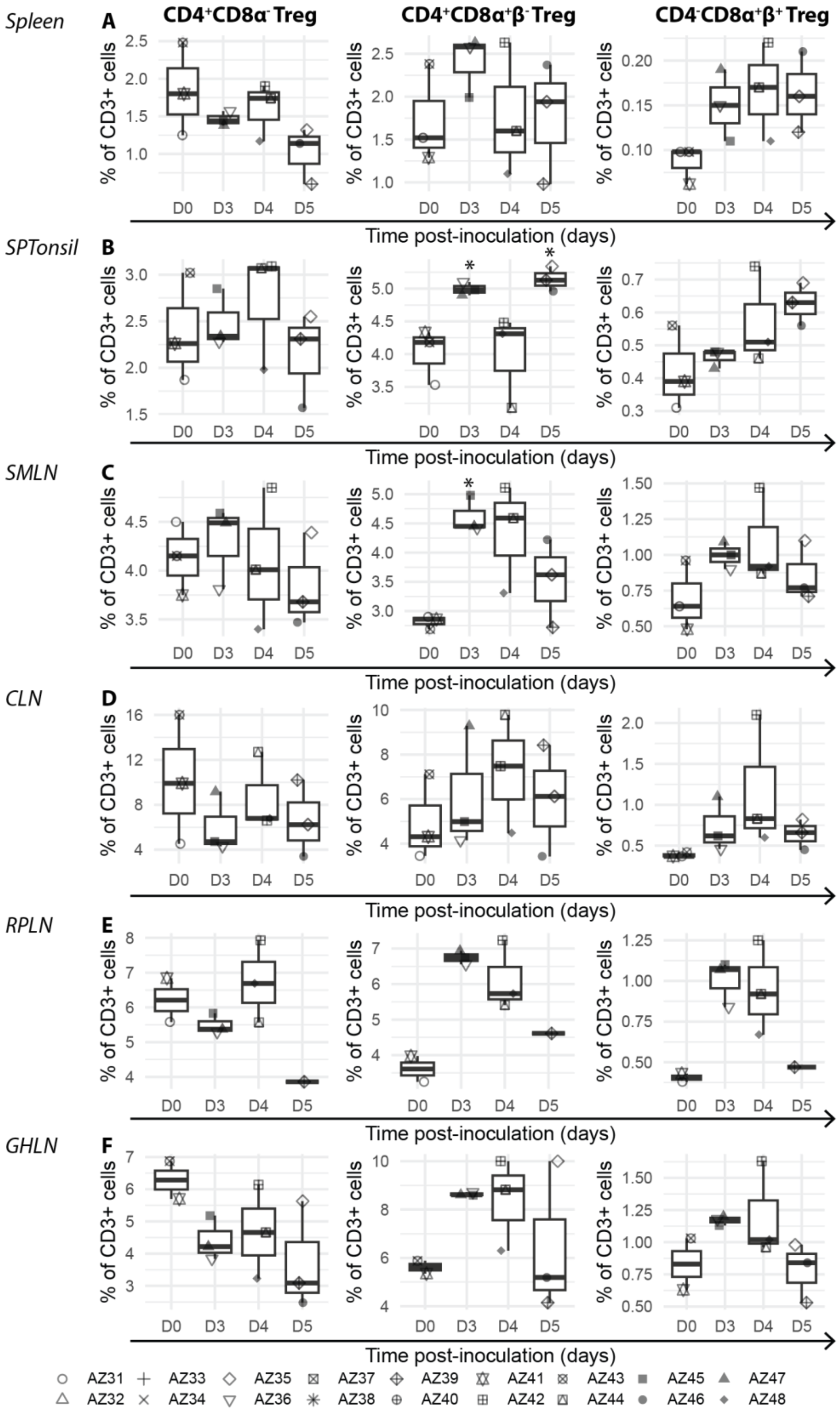

Supplementary Figure 17 Dynamics of $FoxP3^+$ regulatory T cells (Tregs) in various tissues post-inoculation with Georgia 2007/1. Each datapoint denotes a single animal. All tissues have n=3 samples at each timepoint except for RPLN 0 dpi (n=2) and 5 dpi (n=1), and GHLN 0 dpi (n=2). CLN:

cervical lymph node, GHLN: gastro-hepatic lymph node, RPLN: retropharyngeal lymph node, SMLN: submandibular lymph node, SPTonsil: soft palate tonsil. * p<0.05, one way ANOVA, except for (C, $CD4^{+}CD8\alpha^{+}\beta^{-}$ Treg) and (D, $CD4^{-}CD8\alpha^{+}\beta^{+}$ Treg) which used Kruskal-Wallis.

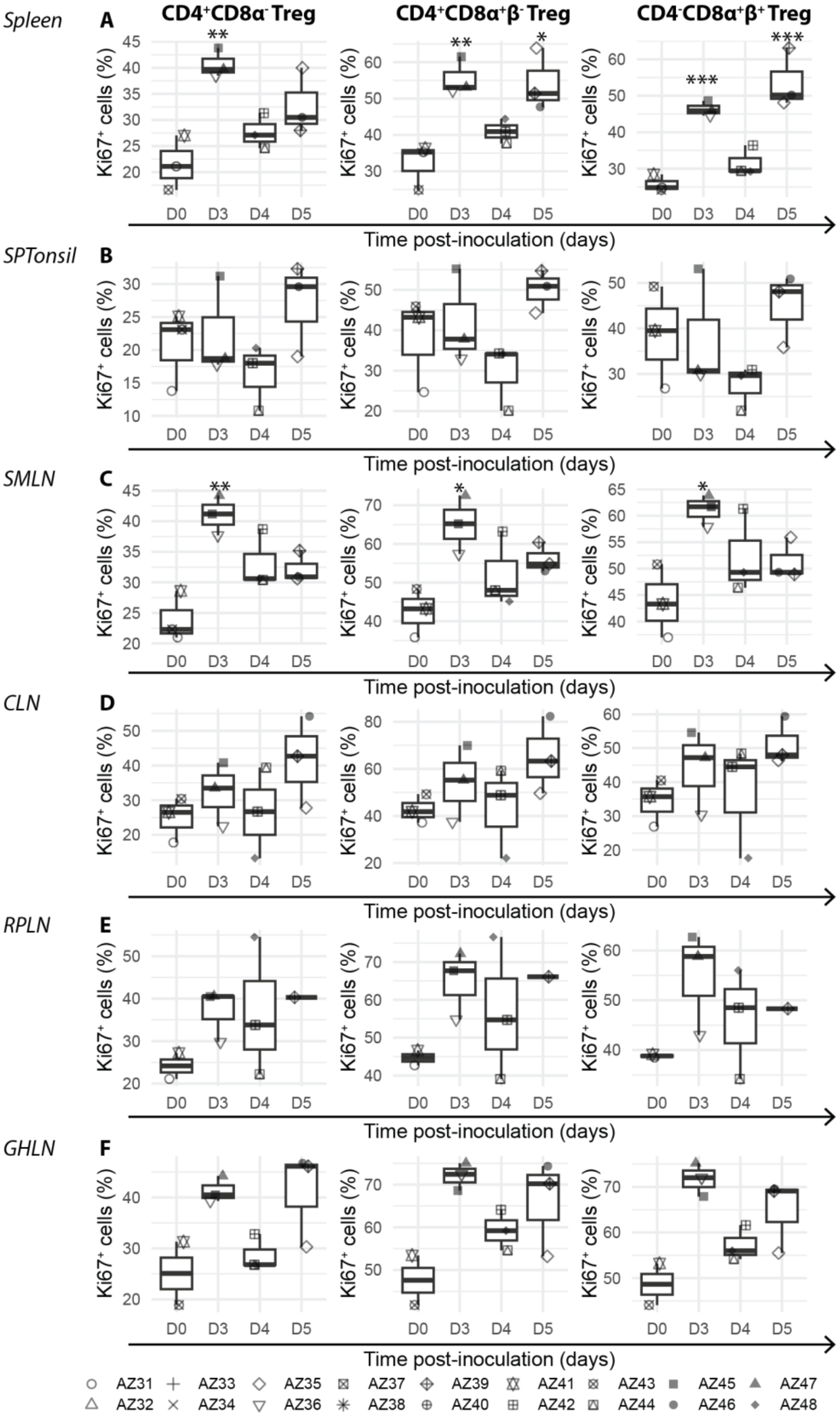

Supplementary Figure 18 Conventional gating CD3+ Treg cell subsets for proliferation in selected tissues post-inoculation with Georgia 2007/1. Each datapoint denotes a single animal. All tissues have n=3 samples at each timepoint except for RPLN 0 dpi (n=2) and 5 dpi (n=1), and GHLN 0 dpi (n=2). CLN: cervical lymph node, GHLN: gastro-hepatic lymph node, RPLN: retropharyngeal lymph

node, SMLN: submandibular lymph node, SPTonsil: soft palate tonsil. * p<0.05, ** p<0.01, *** p<0.001, one way ANOVA.

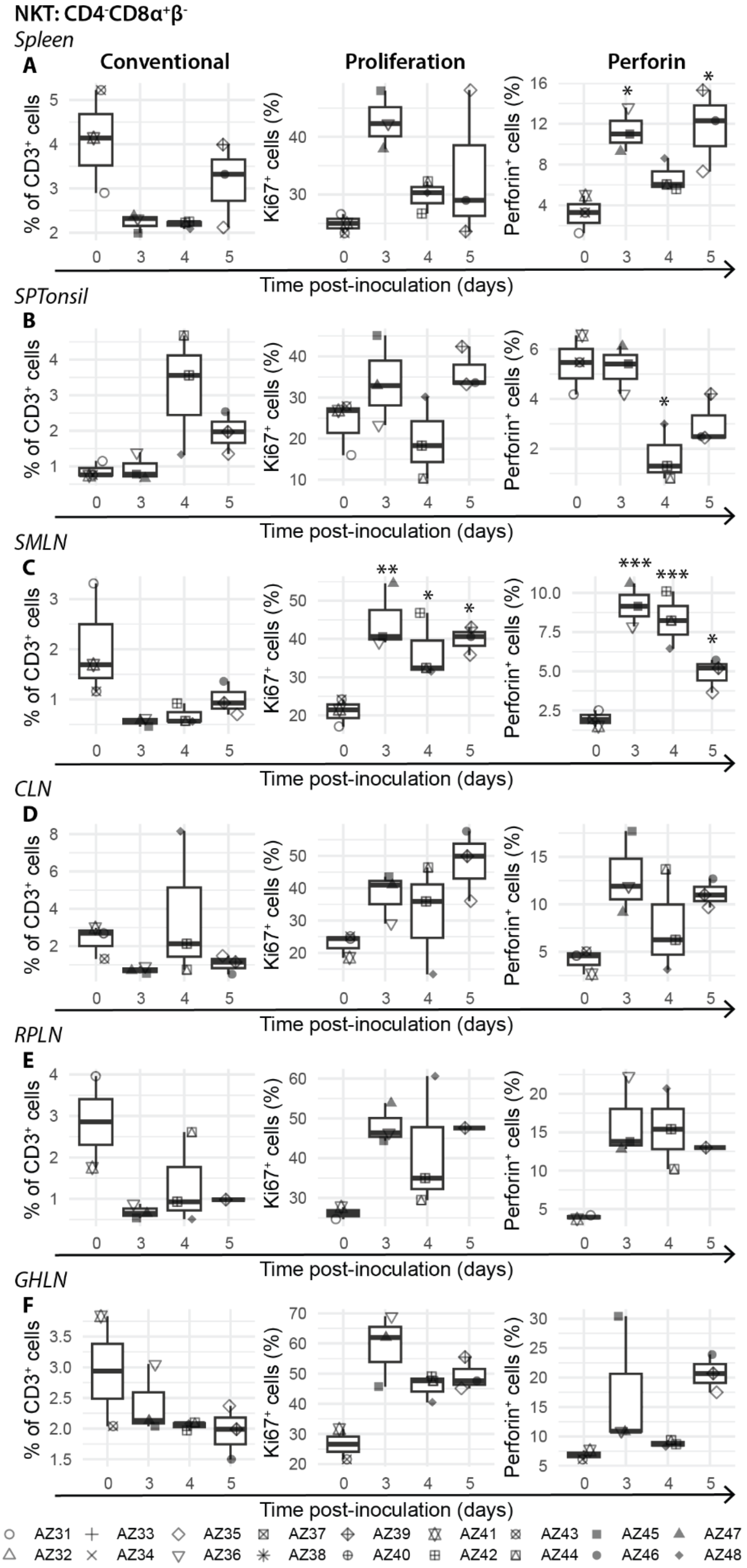

Supplementary Figure 19 Dynamics, proliferation and perforin expression of NKT cells in various tissues post-inoculation with Georgia 2007/1. Each datapoint denotes a single animal. All tissues have n=3 samples at each timepoint except for RPLN 0 dpi (n=2) and 5 dpi (n=1), and GHLN 0 dpi

(n=2). CLN: cervical lymph node, GHLN: gastro-hepatic lymph node, RPLN: retropharyngeal lymph node, SMLN: submandibular lymph node, SPTonsil: soft palate tonsil. * p<0.05, ** p<0.01, *** p<0.001, one way ANOVA except for (A, Conventional) which used Kruskal-Wallis.

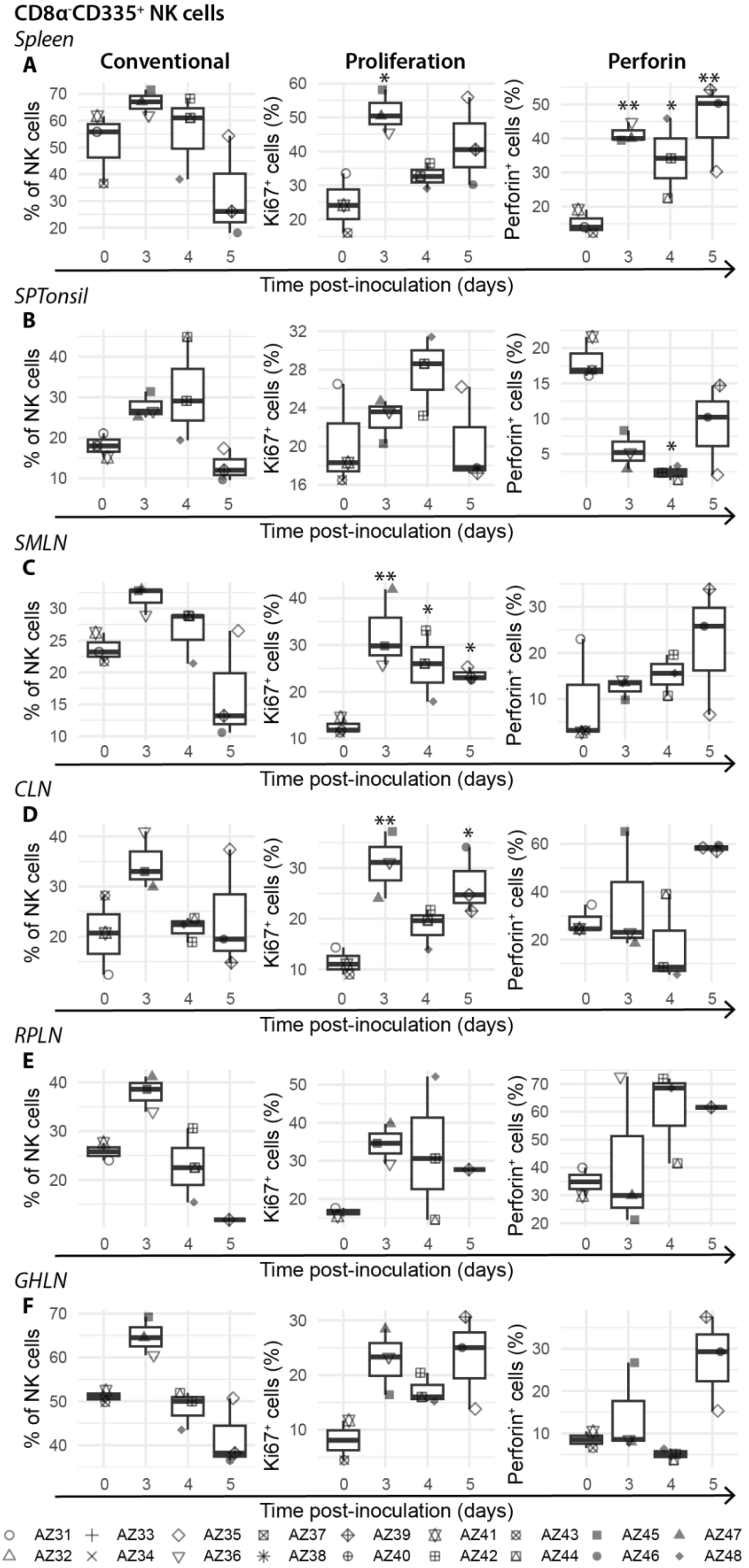

Supplementary Figure 20 Dynamics, proliferation and perforin expression of CD8α$^-$CD335$^+$ NK cells in various tissues post-inoculation with Georgia 2007/1. Each datapoint denotes a single animal.

All tissues have n=3 samples at each timepoint except for RPLN 0 dpi (n=2) and 5 dpi (n=1), and GHLN 0 dpi (n=2). CLN: cervical lymph node, GHLN: gastro-hepatic lymph node, RPLN: retropharyngeal lymph node, SMLN: submandibular lymph node, SPTonsil: soft palate tonsil. * p<0.05, ** p<0.01, one way ANOVA, except for (D, Perforin) which used Kruskal-Wallis.

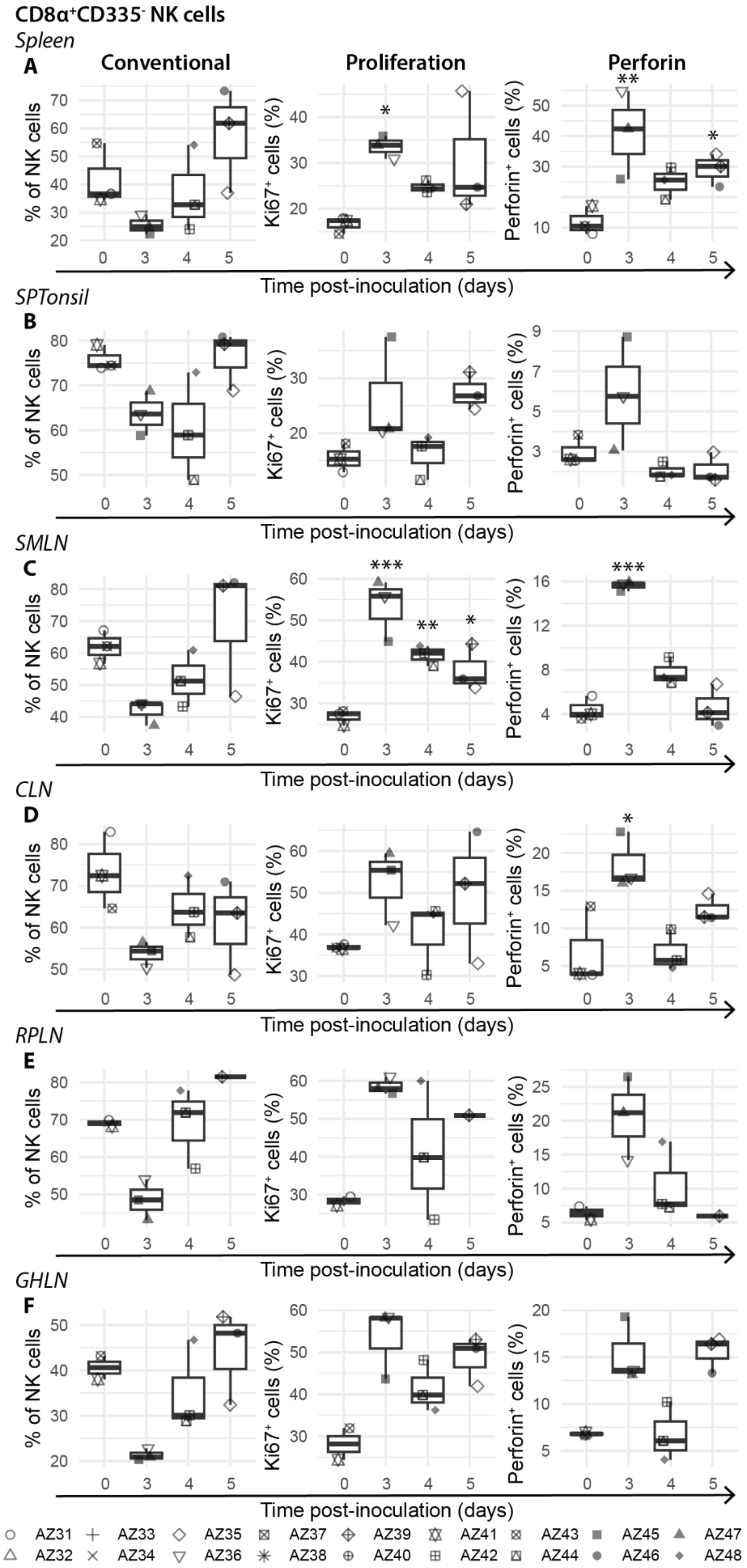

Supplementary Figure 21 Dynamics, proliferation and perforin expression of CD8α^{+}CD335^{-} NK cells in various tissues post-inoculation with Georgia 2007/1. Each datapoint denotes a single animal. All tissues have n=3 samples at each timepoint except for RPLN 0 dpi (n=2) and 5 dpi (n=1), and GHLN 0 dpi (n=2). CLN: cervical lymph node, GHLN: gastro-hepatic lymph node, RPLN: retropharyngeal lymph node, SMLN: submandibular lymph node, SPTonsil: soft palate tonsil. * p<0.05, ** p<0.01, *** p<0.001, one way ANOVA, except for (A, Conventional, Proliferation) and (B, Conventional) which used Kruskal-Wallis.

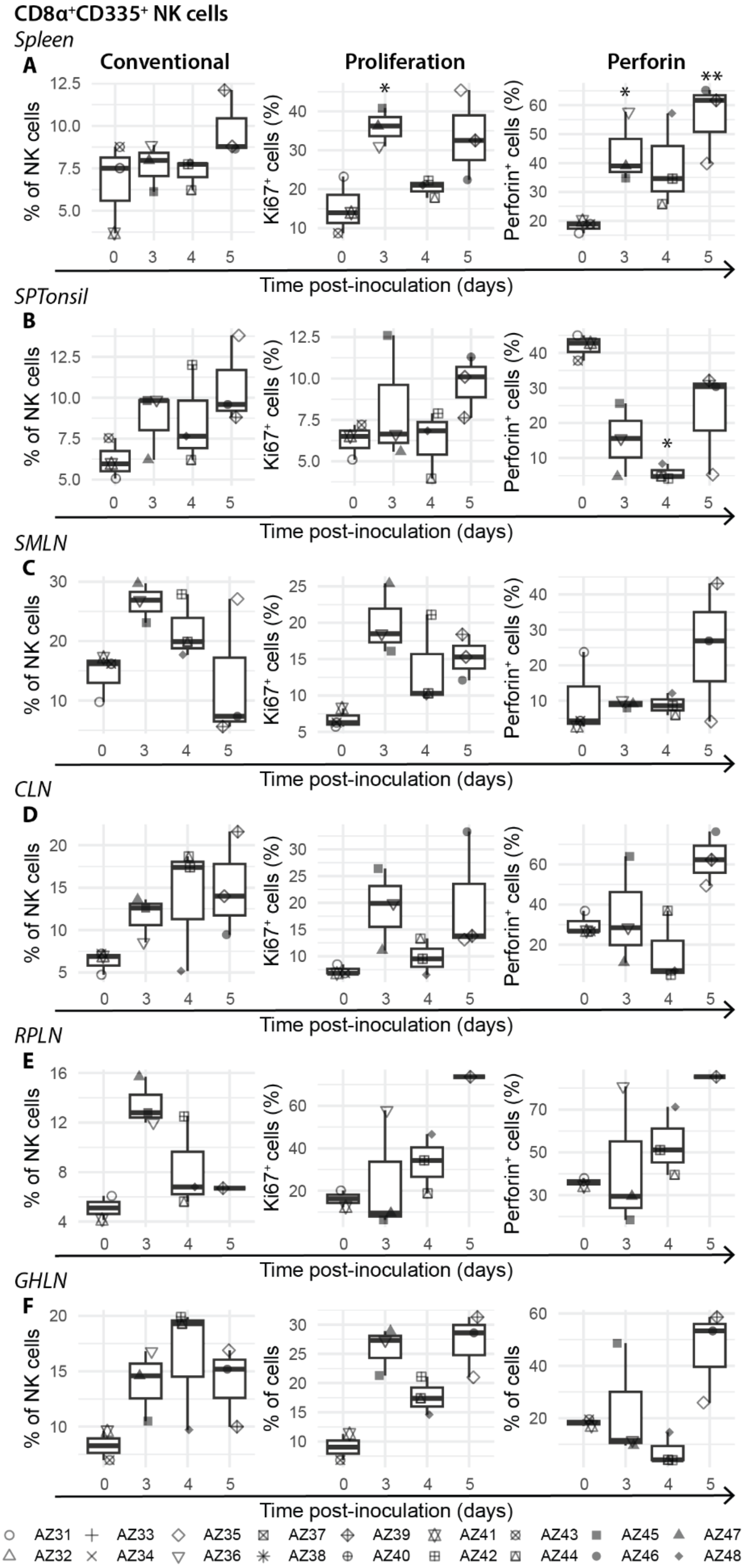

Supplementary Figure 22 Dynamics, proliferation and perforin expression of CD8α⁺CD335⁺ NK cells in various tissues post-inoculation with Georgia 2007/1. Each datapoint denotes a single animal. All tissues have n=3 samples at each timepoint except for RPLN 0 dpi (n=2) and 5 dpi (n=1), and GHLN 0 dpi (n=2). CLN: cervical lymph node, GHLN: gastro-hepatic lymph node, RPLN: retropharyngeal lymph node, SMLN: submandibular lymph node, SPTonsil: soft palate tonsil. *

p<0.05, ** p<0.01, one way ANOVA, except for (B, Conventional) and (D, Conventional) which used Kruskal-Wallis.

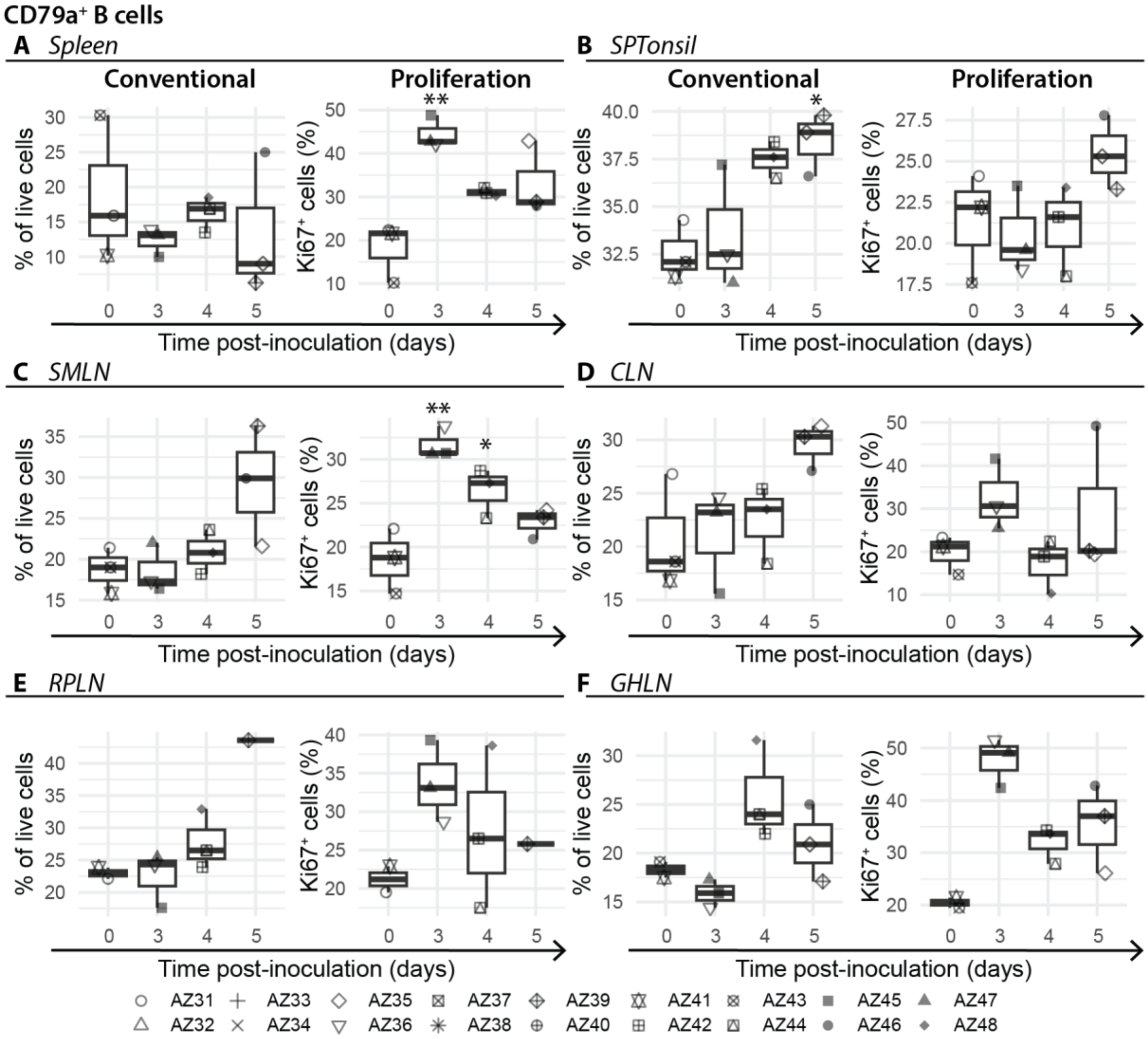

Supplementary Figure 23 Dynamics (Conventional) and proliferation (Proliferation) of B cells in the (A) Spleen, (B) SPTonsil, (C) SMLN, (D) CLN, (E) RPLN and (F) GHLN post-inoculation with Georgia 2007/1. Each datapoint denotes a single animal. All tissues have n=3 samples at each timepoint except for RPLN 0 dpi (n=2) and 5 dpi (n=1), and GHLN 0 dpi (n=2). CLN: cervical lymph node, GHLN: gastro-hepatic lymph node, RPLN: retropharyngeal lymph node, SMLN: submandibular lymph node, SPTonsil: soft palate tonsil. * p<0.05, ** p<0.01, one way ANOVA.

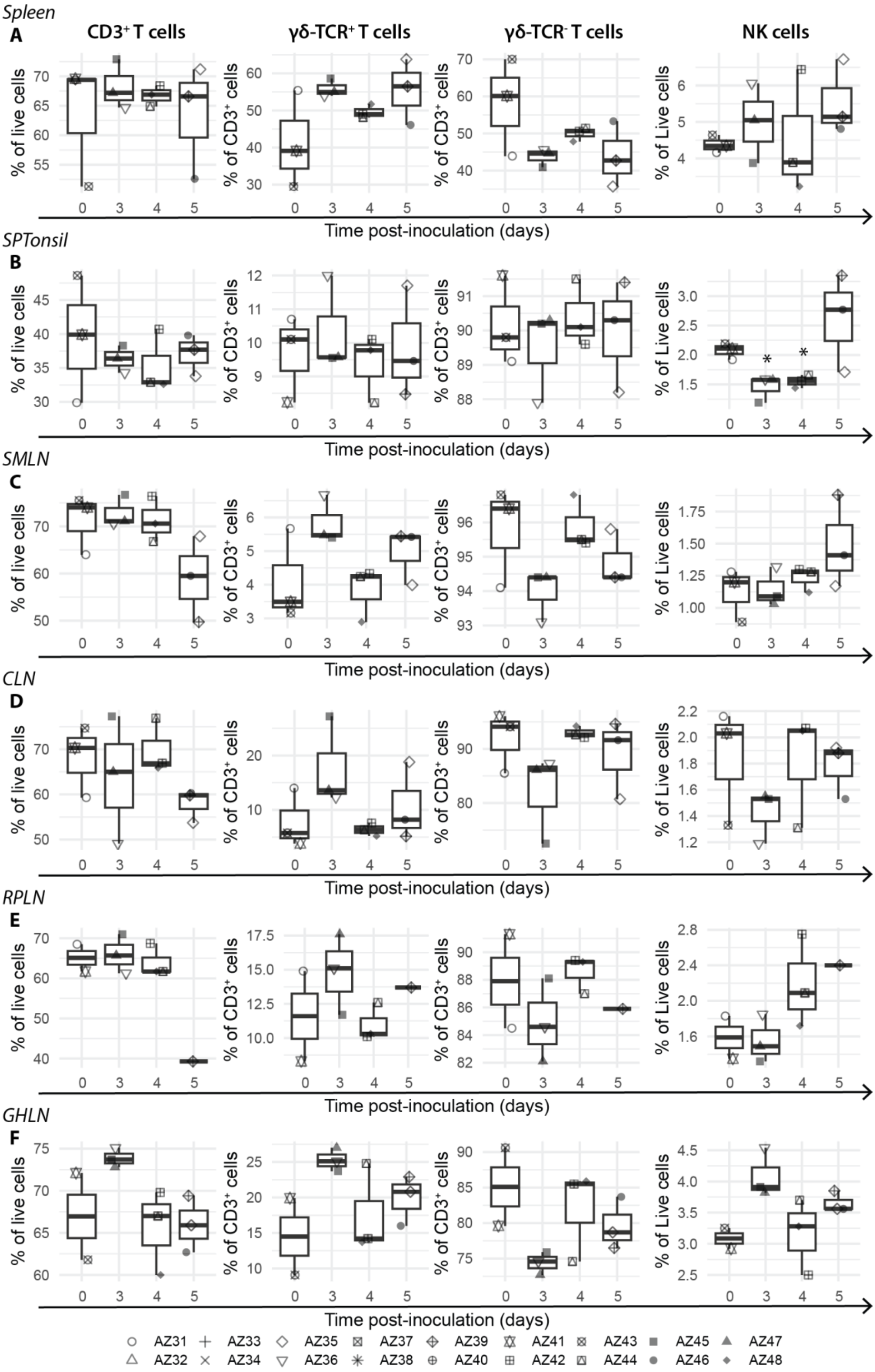

Supplementary Figure 24 Major lymphocyte population dynamics analysed using manual gating in various tissues post-inoculation with Georgia 2007/1. Each datapoint denotes a single animal. All tissues have n=3 samples at each timepoint except for RPLN 0 dpi (n=2) and 5 dpi (n=1), and GHLN 0 dpi (n=2). CLN: cervical lymph node, GHLN: gastro-hepatic lymph node, RPLN: retropharyngeal lymph node, SMLN: submandibular lymph node, SPTonsil: soft palate tonsil. * $p<0.05$, one way

ANOVA, except for (A, CD3⁺ T cells), (C, CD3⁺ T cells) and (D, γδ-TCR⁻ T cells) which used Kruskal-Wallis.

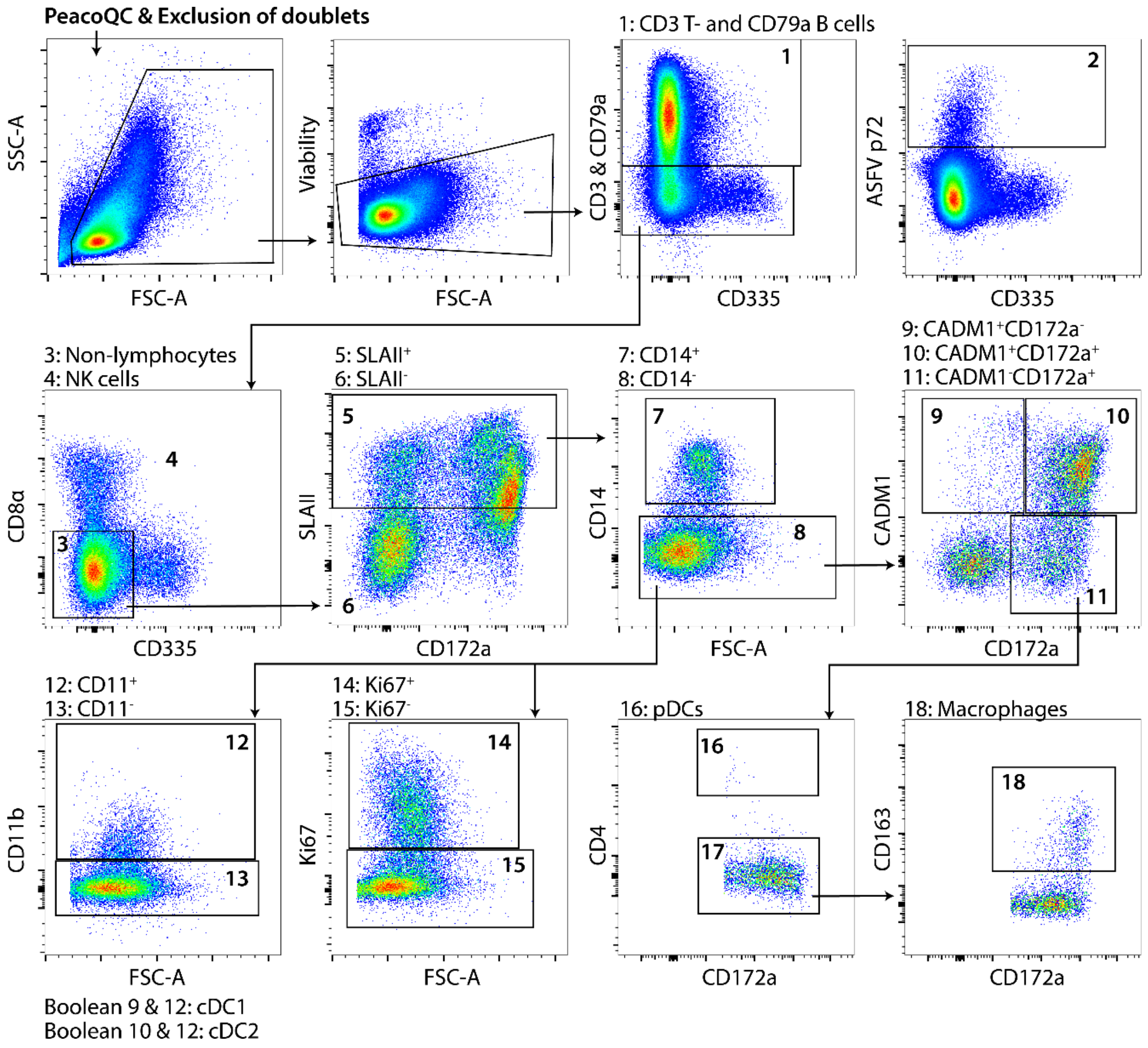

Supplementary Figure 25 Gating strategy for (3) non-lymphocyte tSNE and conventional gating analysis. Representative gating of spleen sample from AZ48 on day 4 post-inoculation with Georgia 2007/1. (1) CD3⁺ T cells and CD79a⁺ B cells, (3) non-lymphocytes after removal of CD3⁻CD8α⁺, CD3⁻CD335⁺ NK cells, (5) SLAII⁺ non-lymphocytes, (7) monocytes, (8) CD14⁻SLAII⁺ non-lymphocytes, (Boolean of 9 and 12) CD11b⁺ cDC1 cells, (Boolean of 10 and 12) CD11b⁺ cDC2 cells, (Boolean of 9 and 13) CD11b⁻ DC1 cells, (Boolean of 10 and 13) CD11b⁻ DC2 cells, (16) pDCs, (17) CD14⁻ macrophage containing population, (18) CD14⁻CD163⁺ macrophages, subsequent Boolean gating with 14 for frequencies of proliferating cells and Boolean gating with (2) for ASFV p72⁺ cells.

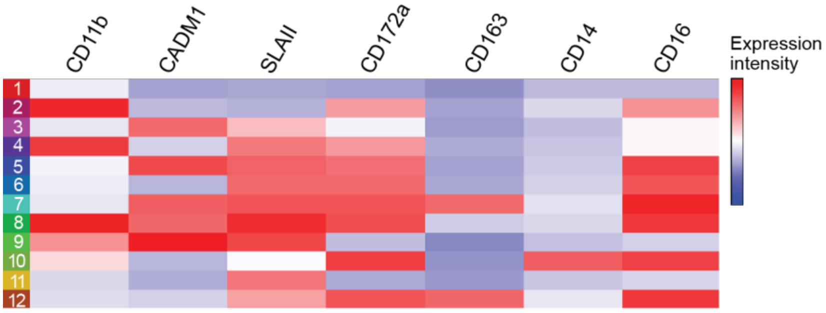

Supplementary Figure 26 Heatmap of clusters identified by FlowSOM on non-lymphocytes subjected to tSNE analysis and the associated marker expression profiles. Numbers on the left column indicate the clusters. Cluster 1: $SLAII^{-}CD172a^{-}$, cluster 2: $SLAII^{-}CD172a^{+}CD11b^{+}CD16^{+}$, cluster 3: $SLAII^{+}CD172a^{-}CADM1^{+}CD11b^{-}$ DC1, cluster 4: $SLAII^{+}CD172a^{+}CD11b^{+}$, cluster 5: $SLAII^{+}CD172a^{+}CADM1^{+}CD11b^{-}CD16^{+}$ DC2, cluster 6: $SLAII^{+}CD172a^{+}CD16^{+}$, cluster 7: $SLAII^{+}CD172a^{+}CADM1^{+}CD163^{+}CD16^{+}$, cluster 8: $SLAII^{+}CD172a^{+}CADM1^{+}CD11b^{+}CD16^{+}$ cDC2, cluster 9: $SLAII^{+}CD172a^{-}CADM1^{+}CD11b^{+}$ cDC1, cluster 10: $CD172a^{+}CD14^{+}CD16^{+}$ monocytes, cluster 11: $SLAII^{+}CD172a^{-}$, cluster 12: $SLAII^{+}CD172a^{+}CD14^{-}CD163^{+}CD16^{+}$ macrophages.

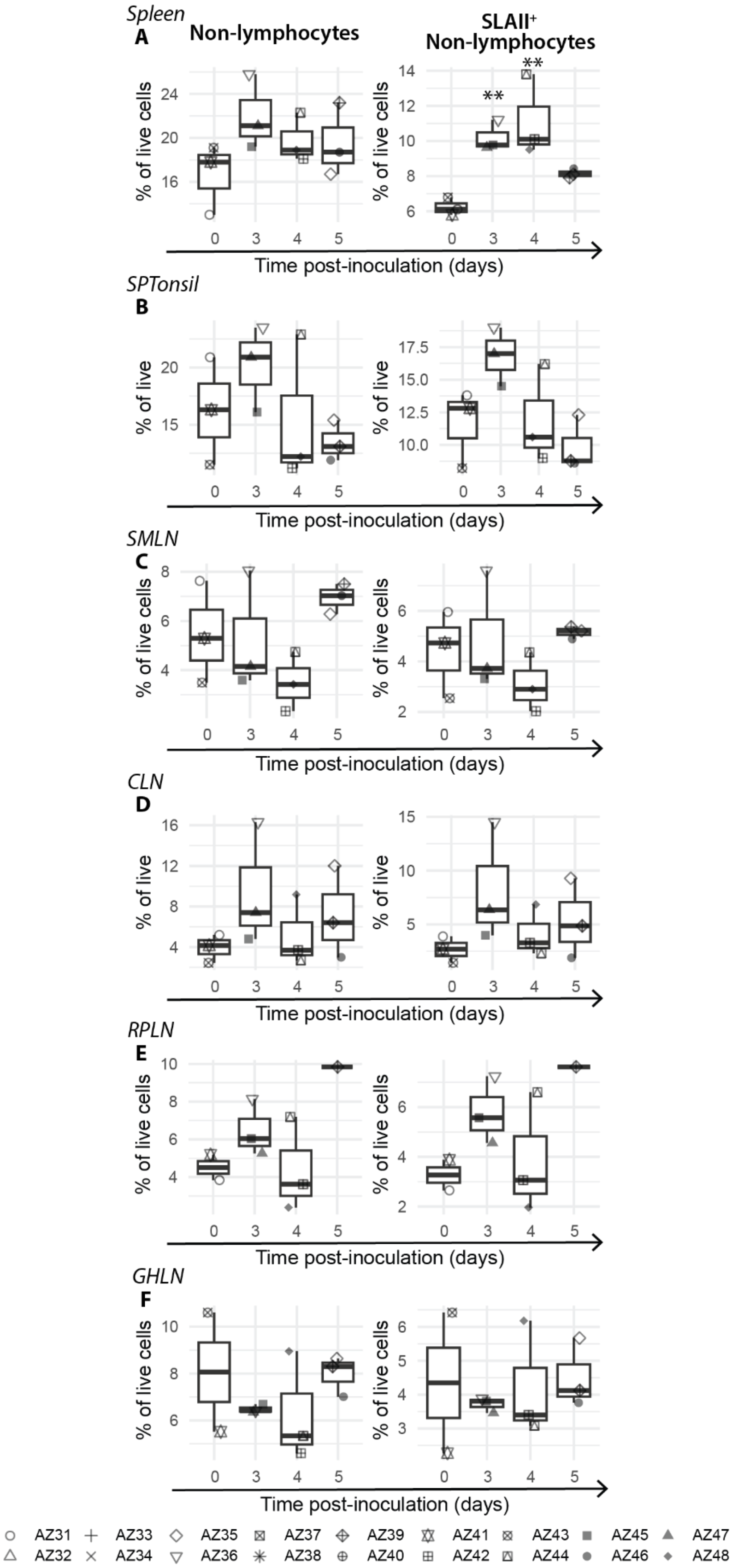

Supplementary Figure 27 Non-lymphocyte population dynamics analysed using manual gating in the (A) spleen, (B) SPTonsil, (C) SMLN, (D) CLN, (E) RPLN and (F) GHLN post-inoculation with Georgia 2007/1. Each datapoint denotes a single animal. All tissues have n=3 samples at each timepoint except for RPLN 0 dpi (n=2) and 5 dpi (n=1), and GHLN 0 dpi (n=2). CLN: cervical lymph node, GHLN: gastro-hepatic lymph node, RPLN: retropharyngeal lymph node, SMLN:

submandibular lymph node, SPTonsil: soft palate tonsil. ** p<0.01, one way ANOVA except for (B, SLAII⁺ non-lymphocytes) which used Kruskal-Wallis.

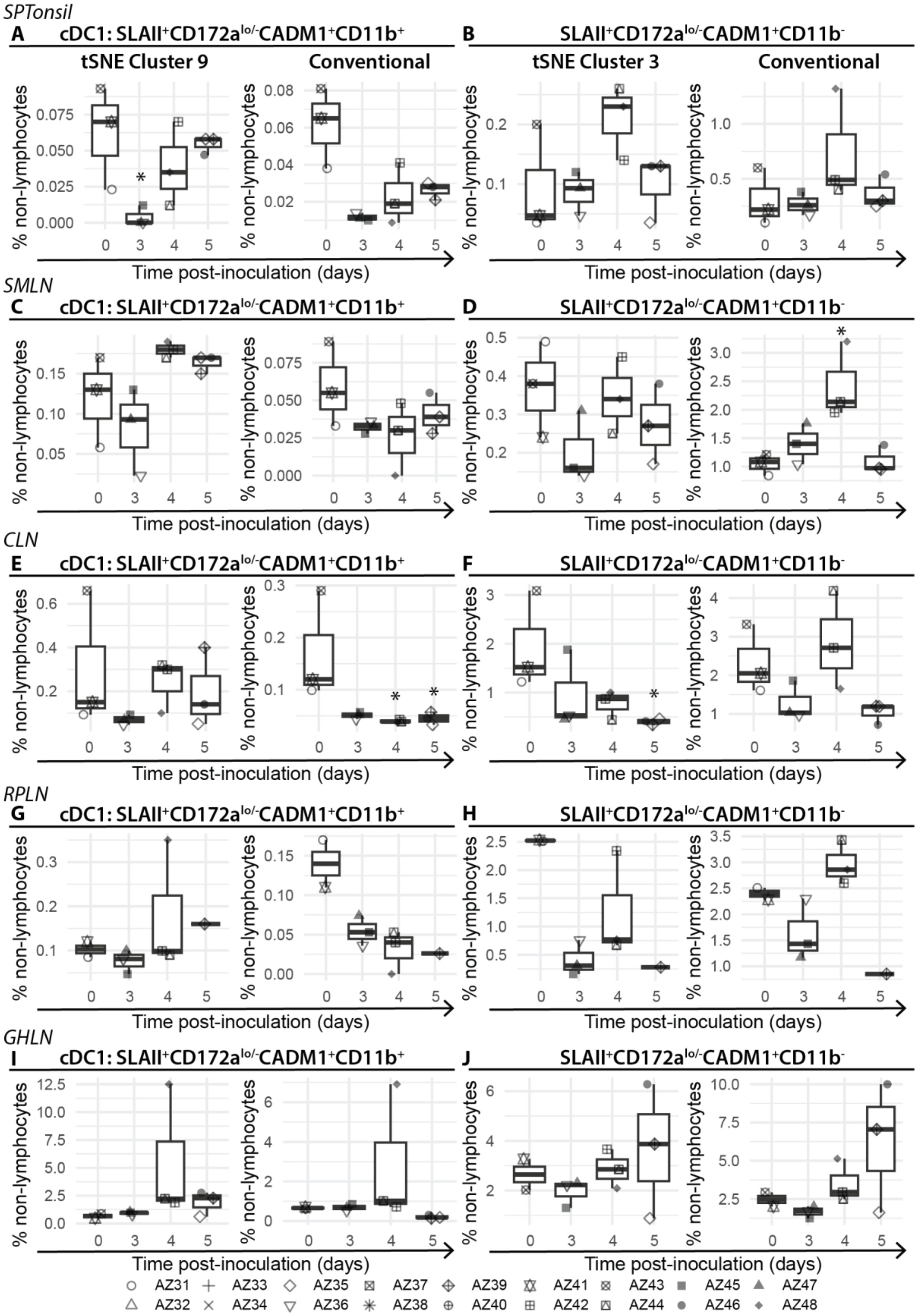

Supplementary Figure 28 Dynamics of dendritic cells, cDC1 and DC1 in the (A,B) SPTonsil, (C,D) SMLN, (E,F) CLN, (G,H) RPLN and (I,J) GHLN post-inoculation with Georgia 2007/1. Each datapoint denotes a single animal. All tissues have n=3 samples at each timepoint except for RPLN 0 dpi

(n=2) and 5 dpi (n=1), and GHLN 0 dpi (n=2). CLN: cervical lymph node, GHLN: gastro-hepatic lymph node, RPLN: retropharyngeal lymph node, SMLN: submandibular lymph node, SPTonsil: soft palate tonsil. * p<0.05, one way ANOVA except for (E, Conventional) which used Kruskal-Wallis.

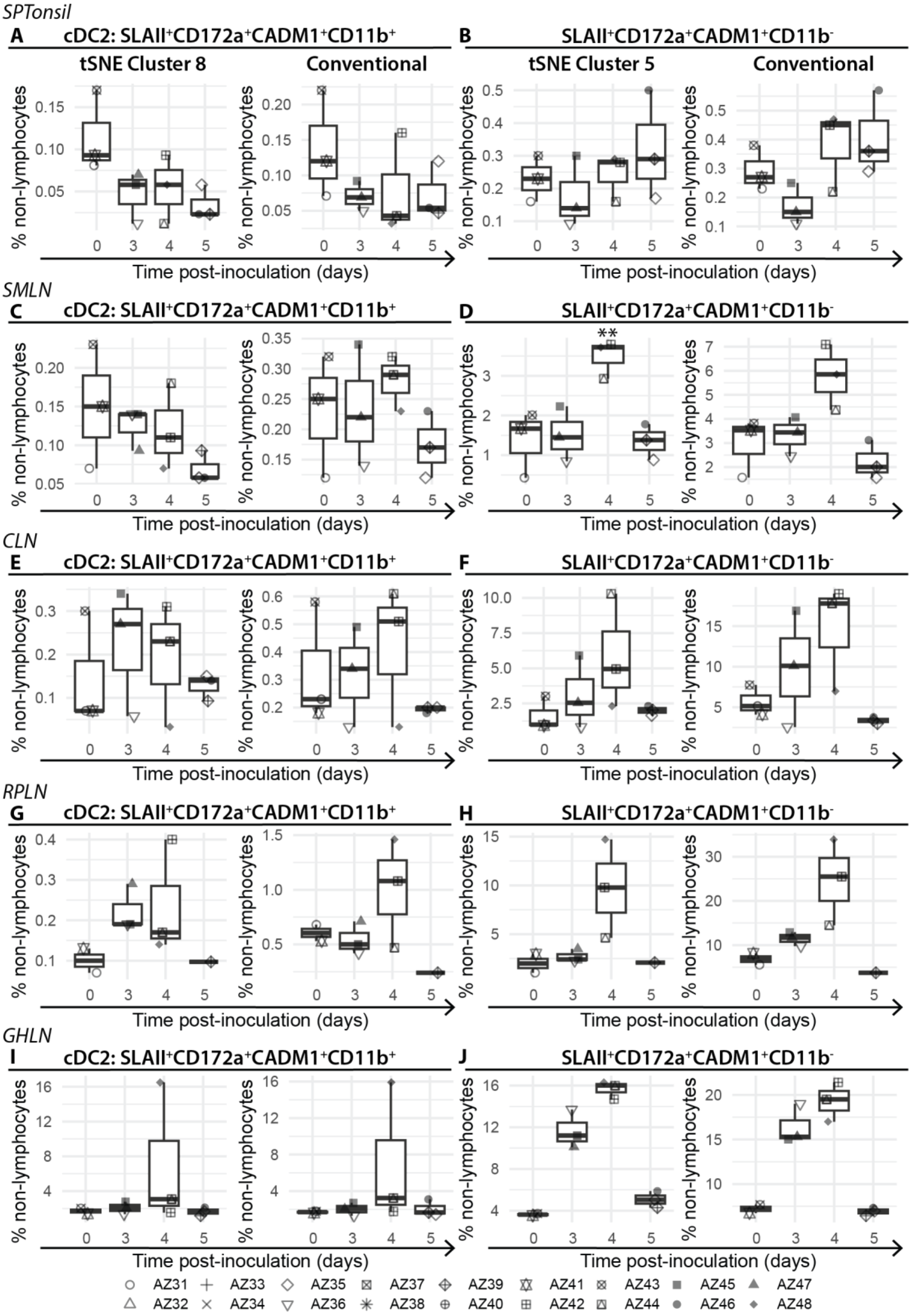

Supplementary Figure 29 Dynamics of dendritic cells, cDC2 and DC2 in the (A,B) SPTonsil, (C,D) SMLN, (E,F) CLN, (G,H) RPLN and (I,J) GHLN post-inoculation with Georgia 2007/1. Each datapoint

denotes a single animal. All tissues have n=3 samples at each timepoint except for RPLN 0 dpi (n=2) and 5 dpi (n=1), and GHLN 0 dpi (n=2). CLN: cervical lymph node, GHLN: gastro-hepatic lymph node, RPLN: retropharyngeal lymph node, SMLN: submandibular lymph node, SPTonsil: soft palate tonsil. ** p<0.01, one way ANOVA except for (F, tSNE Cluster 5, Conventional) which used Kruskal-Wallis.

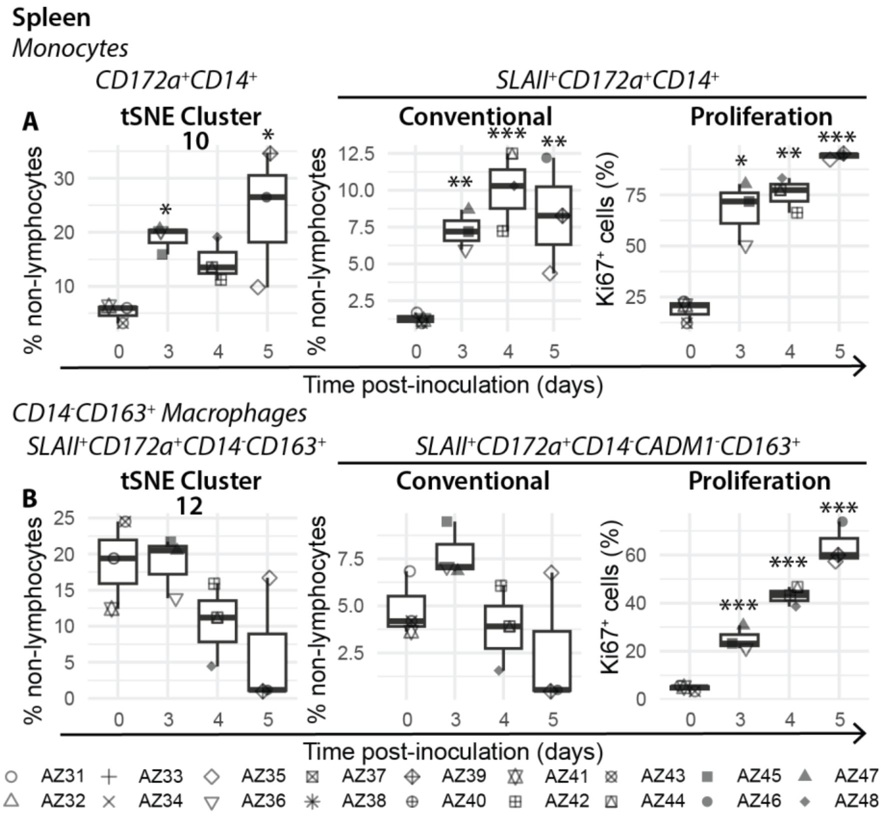

Supplementary Figure 30 Monocyte and $CD14^{-}CD163^{+}$ macrophage population dynamics in the spleen post-inoculation with Georgia 2007/1, using (tSNE Cluster) tSNE and (Conventional) manual gating. (Proliferation) Proliferation of spleen derived monocyte or $CD14^{-}CD163^{+}$ macrophage cell subsets. Each datapoint denotes a single animal. * p<0.05, ** p<0.01, *** p<0.001, one way ANOVA except for (B, Conventional) which used Kruskal-Wallis.

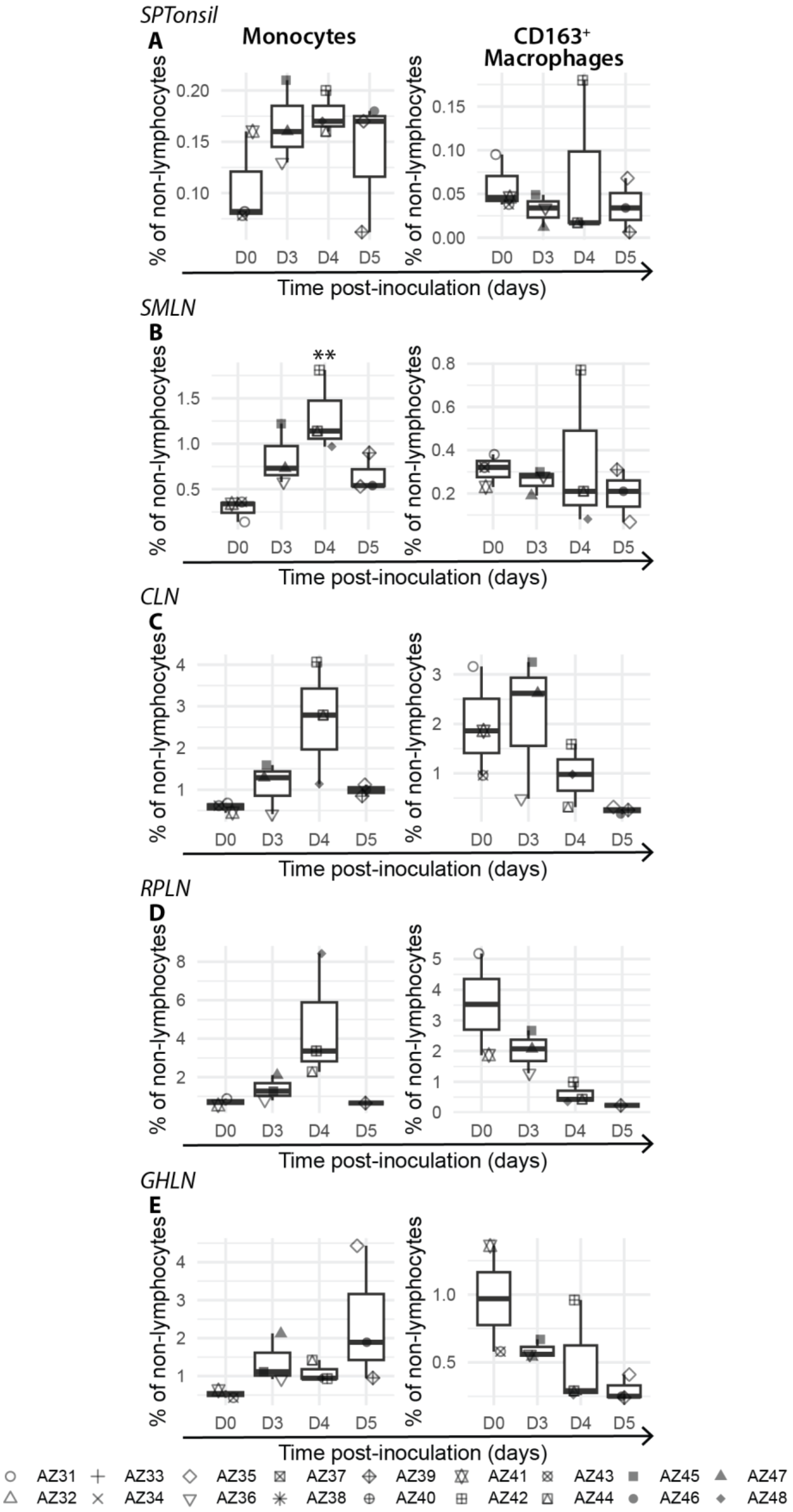

Supplementary Figure 31 Monocyte and $CD14^{-}CD163^{+}$ macrophage population dynamics in various tissues post-inoculation with Georgia 2007/1 and analysed through manual gating. Each datapoint denotes a single animal. All tissues have n=3 samples at each timepoint except for RPLN 0 dpi (n=2) and 5 dpi (n=1), and GHLN 0 dpi (n=2). CLN: cervical lymph node, GHLN: gastro-hepatic

lymph node, RPLN: retropharyngeal lymph node, SMLN: submandibular lymph node, SPTonsil: soft palate tonsil. ** p<0.01, one way ANOVA.

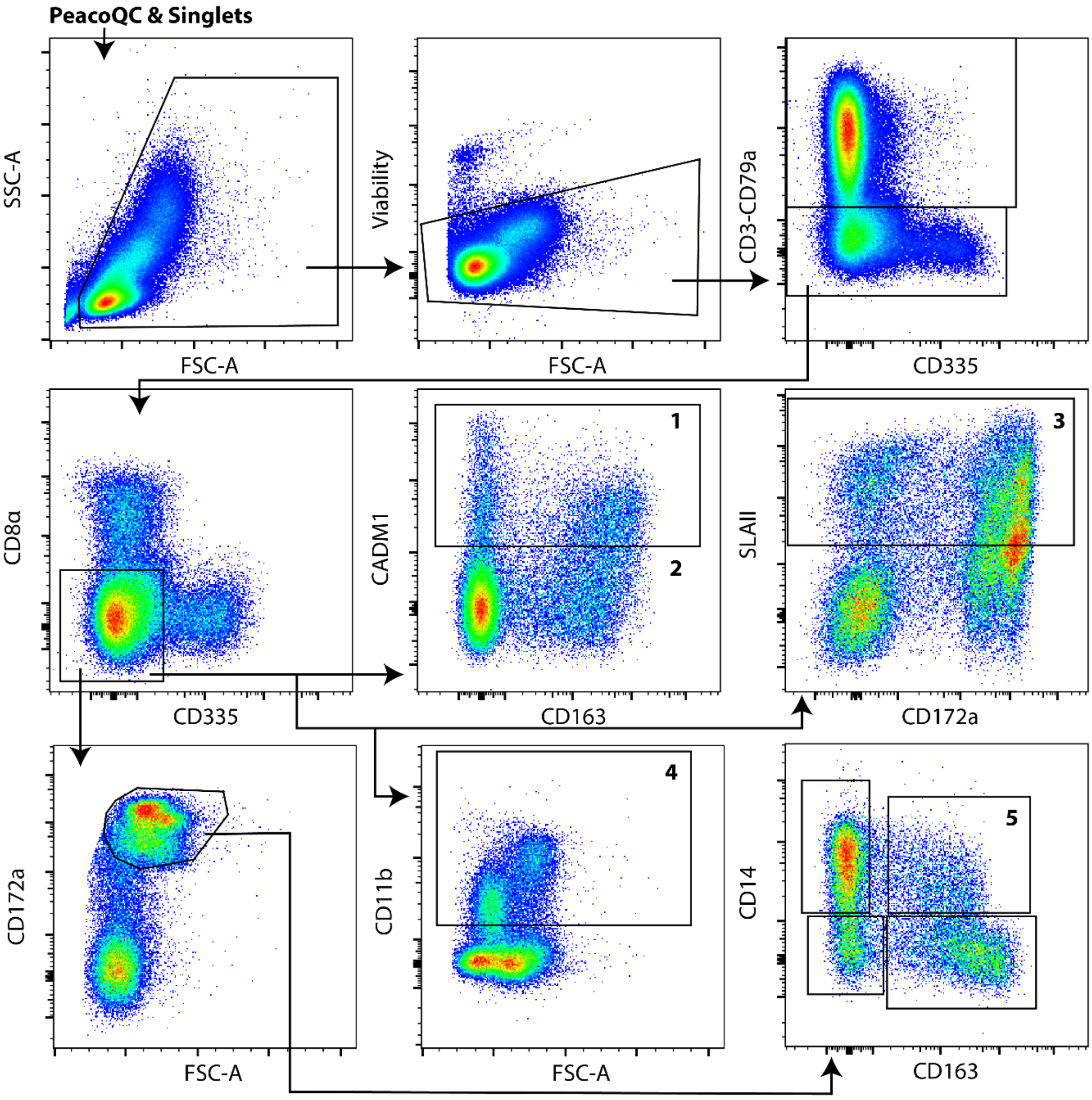

Boolean 3-5: CD14⁺CD163⁺SLAII⁺
Boolean 1, 3-5: CD14⁺CD163⁺SLAII⁺CADM1⁺
Boolean 2, 3-5: CD14⁺CD163⁺SLAII⁺CADM1⁻

Supplementary Figure 32 Conventional gating strategy to identify monocyte derived macrophages. Representative gating of spleen sample from AZ48 on day 4 post-inoculation with Georgia 2007/1.

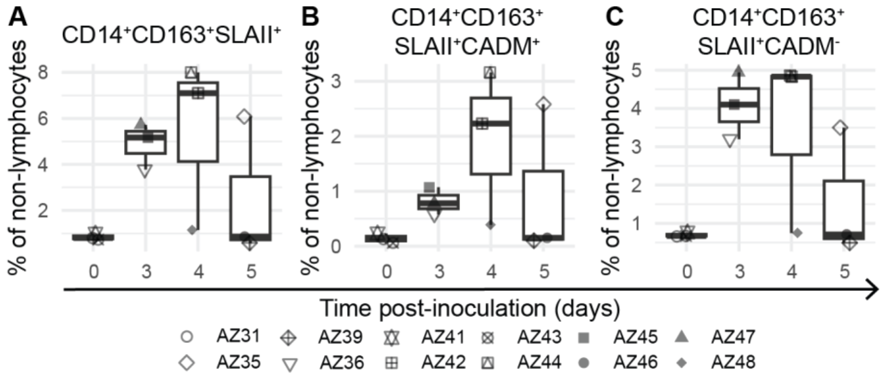

Supplementary Figure 33 Dynamics of monocyte derived macrophages within the spleen post-inoculation with Georgia 2007/1. Each datapoint represents a single animal. One way ANOVA.

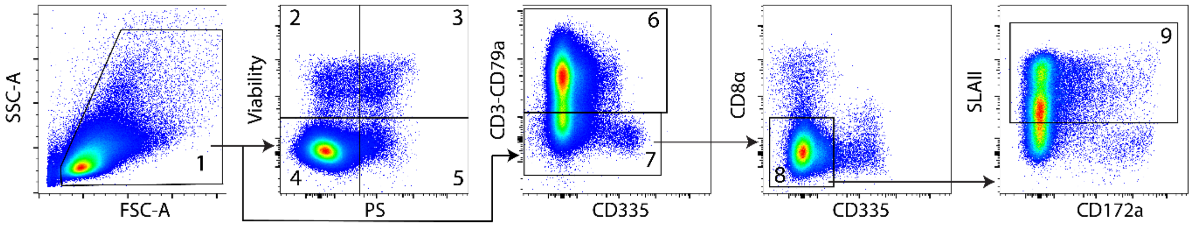

Supplementary Figure 34 Gating strategy for the fixed apoptosis necrosis assay. Representative gating of SPTonsil sample from AZ44 on day 4 post-inoculation with Georgia 2007/1.

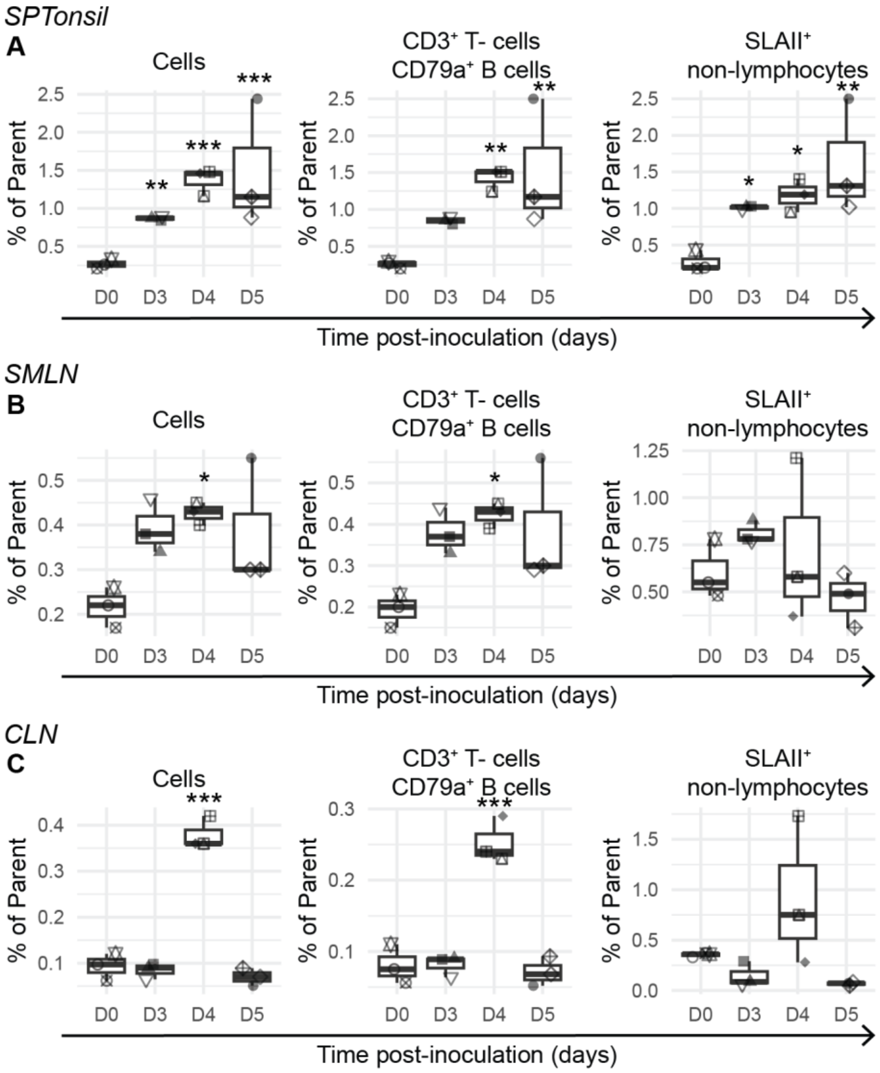

Supplementary Figure 35 Early apoptosis of major cell compartments in lymphoid tissues draining the early sites of replication. Each datapoint denotes a single animal. CLN: cervical lymph node, SMLN: submandibular lymph node, SPTonsil: soft palate tonsil. * $p<0.05$, ** $p<0.01$, *** $p<0.001$, one way ANOVA.

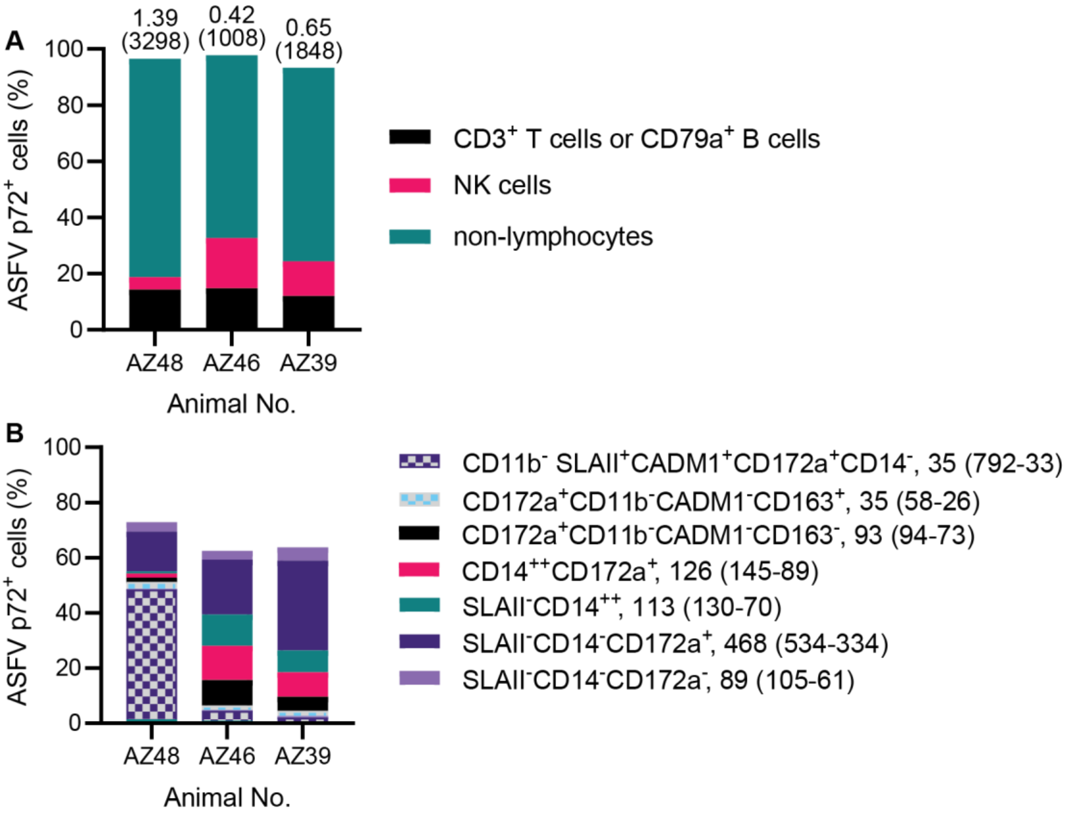

Supplementary Figure 36 Proportion of $p72^+$ cells detected by flow cytometry in the spleens of the three animals with the highest spleen viral loads that are from the (A) major lymphocyte and non-lymphocyte compartments and (B) phenotyped non-lymphocyte subsets. (A) Numbers above each bar indicate the frequency of $p72^+$ cells detected within the live population. Numbers within brackets denote the counts of $p72^+$ cells within each spleen sample. (B) Median counts, (IQR) of each phenotype is listed with each subset.

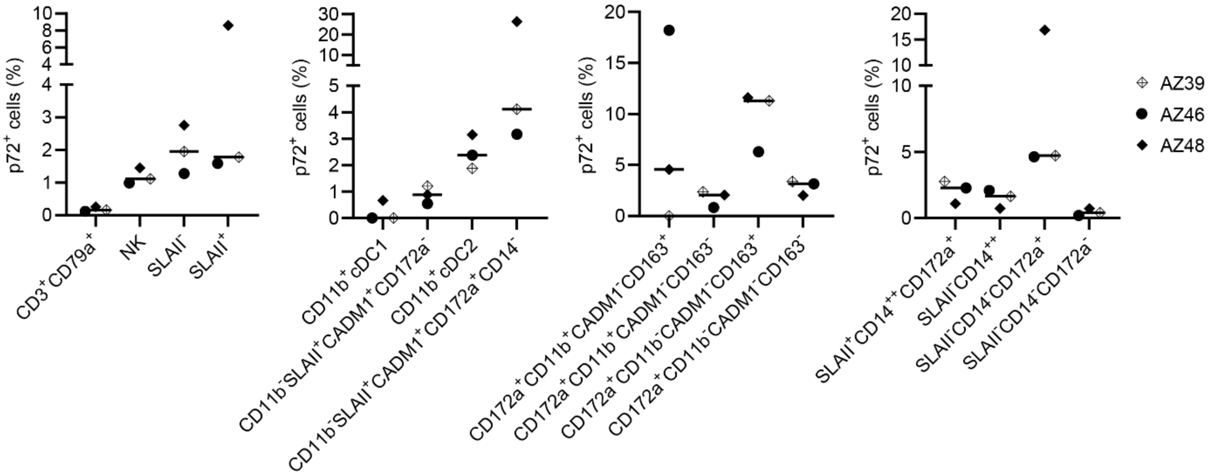

Supplementary Figure 37 Susceptibility of selected cell subsets to ASFV infection as detected by the presence of ASFV p72 with flow cytometry. Each datapoint denotes a single animal.

FlowJo Packages used

1. PeacocQC FlowJo package: Emmaneel A, Quintelier K, Sichien D et al. Peacoqc: Peak-based selection of high quality cytometry data. Cytometry A 2022;101:325-38. https://doi.org/10.1002/cyto.a.24501

2. FlowSOM clustering: Van Gassen S, Callebaut B, Van Helden MJ et al. Flowsom: Using self-organizing maps for visualization and interpretation of cytometry data. Cytometry A 2015;87:636-45. https://doi.org/10.1002/cyto.a.22625

R and Packages used

1. R (version 4.4.0) R Core Team. R: A language and environment for statistical computing. *R Foundation for Statistical Computing* 2024. http://https://www.R-project.org/
2. RStudio (version 2023.9.1.494) Posit team. Rstudio: Integrated development environment for r. *Posit Software, PBC* 2023. http://http://www.posit.co/
3. multcomp package: Hothorn T, Bretz F, Westfall P. Simultaneous inference in general parametric models. *Biom J* 2008;**50**:346-63. https://doi.org/10.1002/bimj.200810425
4. dunn.test package: Dinno A. Dunn.Test: Dunn's test of multiple comparisons using rank sums. 2024. http://https://CRAN.R-project.org/package=dunn.test
5. ggplot2 package: Wickham H. *Ggplot2: Elegant graphics for data analysis*. Springer-Verlag New York}, 2016.
